# *Aspergillus flavipes* L-methionine γ-lyase-β-cyclodextrin conjugates with improved stability, catalytic efficiency and anticancer activity

**DOI:** 10.1038/s41598-024-78368-5

**Published:** 2024-11-12

**Authors:** Ashraf S. A. El-Sayed, Ahmed Shindia, Esraa Emam, Mai Labib, Eman Nour El-Deen, Mohamed G. Seadawy, Marwa A. Yassin

**Affiliations:** 1 Enzymology and Fungal Biotechnology Lab, Botany and Microbiology Department, Faculty of Science, Zagazig University, Zagazig, 44519 Egypt; 2 Agriculture Genetic Engineering Research Institute (AGERI), Agricultural Research Center, Giza, 12619 Egypt; 3 Histopathology Department, Faculty of Medicine, Zagazig University, Zagazig, 44519 Egypt; 4 Biological Prevention Department, Egyptian Ministry of Defense, Cairo, Egypt

**Keywords:** *Aspergillus flavipes*, L-Methionine γ-lyase, Cyclodextrin conjugation, Enzymes, Biochemistry, Biotechnology, Drug discovery, Microbiology

## Abstract

*Aspergillus flavipes* L-methionine γ-lyase (MGL) has been authenticated as a powerful anticancer agent towards various solid tumors, however, the catalytic efficiency and stability of this enzyme remains the main challenge for its further in vivo applications. Thus, the objective of this study was to enhance the catalytic efficiency, structural stability of *A. flavipes* MGL, in addition to boost their anticancer activity*,* via conjugation with β-cyclodextrin. The purified *A. flavipes* MGL was (38.1 μmol/mg/min) was conjugated with β-cyclodextrin, with immobilization yield 80%. The conjugation process of MGL with β-cyclodextrin was verified from the FTIR analysis, molecular docking analysis, ensuring the covalent conjugation process via the hydrogen, and hydrophobic interactions with the cyclodextrin hydroxyl groups and MGL surface amino acids residues. The free and CD-MGL have the same optimum reaction temperature 37 °C, reaction pH 7.5 and pH stability pH 6.5–8.0. The CD-MGL conjugates had a significant stability to proteinase K and trypsin digestion. The affinity of CD-MGL was increased by ~ 2 folds to L-methionine (*K*_M_ 3.1 mM), compared to the free one (*K*_M_ 7.2 mM), as well as the catalytic efficiency of MGL was increased by 1.8 folds upon cyclodextrin conjugation. The higher affinity of CD-MGL for L-methionine might be due to re-orientation of the MGL to bind with the substrate by multiple interactions hydrogen, hydrophobic and covalent bonds compared to the free one. The thermal stability of MGL was increased by ~ 2 folds for the tested treatments, upon cyclodextrin conjugation. The in vitro anticancer activity of CD-MGL was enhanced by 2 folds against the HCT-116 (IC_50_ value 13.9 μmol/mg/min) and MCF7 (IC_50_ value 9.6 μmol/mg/min), compared to the free MGL (~ 21.4 μmol/mg/min). The enzymes displayed a significant activity against the proliferation of Ehrlich ascites carcinoma in vivo, with an obvious improvement on the liver tissues, as revealed from the histopathological sections

**Supplementary Information:**

The online version contains supplementary material available at 10.1038/s41598-024-78368-5.

## Introduction

L-Methionine γ-lyase (MGL, E.C. 4.4.1.11) is a pyridoxal 5’-phosphate dependent enzyme catalyzing the γ-elimination reactions of L-methionine to methanethiol, α-ketobutyrate and ammonia^1,2^. This enzyme has been recognized with its powerful activity against the proliferation of methionine-dependent tumors by methionine depletion, halting their cellular proliferation, causing prompt cell apoptosis^3,4^. The antitumor rationality of MGL elaborates from the lack of active methionine synthase in tumor cells “methionine auxotrophic”^5,6^, making the tumor cells are absolutely dependent on plasma L-methionine for their rapid proliferation, in contrary to the normal cells that have active methionine synthase, ensuring the targetability of MGL for attacking the tumor cells only^5–8^. MGL is a non-mammalian enzyme, however, several human microbial flora were reported to produce this enzyme as reviewed in^9^. MGL is a homotetrameric enzyme, with four pyridoxal 5’-phosphate (PLP) moieties and subunit molecular structure of 50 kDa^1,10,11^. The MGL has been cloned from various bacterial isolates especially *Pseudomonas putida*^1,12–15^, displayed a powerful activity against broad-range of methionine-dependent solid tumors. Although the promising anticancer activity of bacterial MGL, however, the antigenicity, biological short half-life time, and structural un stability are the main limiting factors that halts their further therapeutic applications^15,16^. Remarkably, the MGL from the fungal source “*Aspergillus flavipes*” exhibited an affordable biochemical properties such as structural stability, and lower antigenicity than bacterial MGL^9,17–22^, that could be ascribed to the biochemical compatibility with human, due to the higher similar biological identity of mammalian cells and fungal cells. However, the catalytic efficiency of *A. flavipes* MGL remains the challenges. We have motivated for implementing several approaches for increasing the catalytic efficiency of *A. flavipes* MGL via conjugation with polyethylene glycol and dextran^17,21^, that showed a great improving on the catalytic efficiency of this enzyme. However, the inhibition of the enzyme activity, partial enzyme denaturation, low immobilization yields of *A. flavipes* MGL covalently conjugated with polyethylene glycol and dextran, are the main limitations, that could be due to the interaction of the chemical linkers with the enzyme active sites^17,19–21,23–26^. So, exploring of novel biocompatible compounds for improving the catalytic properties and prolonging the biological half-life time of *A. flavipes* MGL was our objective.

Recently, cyclodextrins are one of the most authenticated biocompatible polymers that have been used in drug formulation and delivery, for their capacity of stabilizing the tertiary structures of proteins against denaturation, low cost and low toxicity that broadened its applications^27^. Cyclodextrins are cyclic oligosaccharides with 6, 7, or 8 gluco-pyranose units with hydrophobic interiors, referred as α-, β-, or γ-, cyclodextrins, respectively. β-Cyclodextrin (β-CD) is a cone-shaped molecule, with hydrophilic outer surface due to the frequent hydroxyl groups, and hydrophobic in the cavity^28,29^. Due to these characteristic features, β-CD has been frequently applied in the drug-delivery, and controlled release, the lipophilic drugs of a size compatible with the hydrophobic core of a cyclodextrin can form complexes, resulting in increasing the aqueous solubility of the target drug^30,31^. Thus, the objective of this study was to enhance the catalytic efficiency, stabilizing the structural stability, and increasing the anticancer activity of MGL purified from *A. flavipes*, upon β-cyclodextrin conjugation.

## Materials and methods

### Materials

Pyridoxal 5’-phosphate, DL-homocysteine, L-methionine, β-mercaptoethanol, hydroxylamine, iodoacetate, guanidine thiocynate and 5,5-dithiobis (2-nitrobenzoic acid) were obtained from Sigma-Aldrich Co. (MO, USA). 3-Methyl-2-benzo-thiazolinonhydrazone hydrochloride was obtained from Merk Co. (Darm., Germany). All the other chemicals were of analytical grade.

### *Aspergillus flavipes* nutritional bioprocessing by the Plackett–Burman Design for maximum MGL productivity

*Aspergillus flavipes* (JF831014) as the potent MGL producer from our studies^9,17–20^ has been grown on modified Dox’s medium of 1% L-methionine for 8 days at 30 °C at 120 rpm^19,20^. After incubation, the cultures were filtered, the fungal pellets (10 g) were washed with sterile distilled water and intracellular crude proteins were extracted by grinding in liquid nitrogen, dispensed in potassium phosphate buffer (50 mM, pH 7.8) and 10 μM PLP^32,33^. The homogenate was centrifuged for 10 min at 8000 rpm, and the supernatant was used for the enzyme assay and protein concentration^9,20,34^.

The MGL productivity by *A. flavipes* has been optimized by the Response Surface Methodology with the two-factorial Plackett–Burman Design and Faced Central-Composite design (CCD). The selected physicochemical parameters “L-methionine, glycine, valine, L-asparagine, L-glutamine, L-lysine, D-glucose, fructose, xylose, sucrose, MgCl_2_, ZnCl_2_, Vitamin B6, shaking speed, pH, and incubation time” were optimized for maximizing the MGL productivity by *A. flavipes*. The tested nineteen variables were initially screened by the Plackett–Burman design, each variable was exemplified by high (+ 1) and low (− 1) levels. The model of the Plackett–Burman design was follows the first order reaction:

$${\text{Y }} = \, \beta 0 \, + \Sigma \beta {\text{iXi}}$$

Y is the predicted activity of enzyme, Xi is the independent variable, βi is the linear coefficient, and β0 is the model intercept. The runs were conducted in triplicates, and the response was represented by the average of MGL activity. After incubation, the fungal cultures were collected, and the intracellular proteins were extracted, and the enzyme activity was assessed ^17,18^.

### Purification and molecular subunit structure of MGL from *A. flavipes*

*Aspergillus flavipes* was grown on the optimized medium from the Response surface Methodology bioprocessing. After incubation, the fungal pellet were collected, washed by saline solution prior to intracellular crude proteins extraction^19,21,35^. One hundred grams of the fungal pellets were pulverized in liquid N_2_, dispensed in 100 mL Tris–HCl buffer (pH 7.0, 10 mM) with 1 mM PMSF, 1 mM EDTA and 10 μM PLP^25,26^. The enzyme preparation was fractionally concentrated with 20 kDa cut-off dialyzer (20 kDa, 546–00051) against polyethylene glycol 6000 till minimum volume (~ 25 mL). The crude protein was precipitated by chilled acetone (1:1 v/v) for 15 min at − 20 °C, then the pellets were dissolved in 2 mL Tris–HCl (pH 7.0, 10 mM) with 10 μM PLP. The enzyme was purified by gel filtration chromatography with Sephadex G_100_ column^17,19,23,25,33,36^. After column equilibration by Tris–HCl buffer (pH 7.0, 10 mM), the enzyme was loaded, and eluted by the same buffer, with the flow rate 1 mL/min. The activity and protein concentration of the collected fractions were determined. The enzyme was purified by the ion-exchange chromatography by DEAE-Sepharose column. The enzyme was loaded to the column, eluted with Tris–HCl (pH 7.5, 10 mM) of gradient NaCl concentrations (zero, 50, 100, and 200 mM). The most active and molecularly homogenous fractions were gathered in presence of 1% Tween 20, and the fractions were concentrated using 30 kDa ultracentrifugal membrane, for endotoxins removal^37,38^. The concentrated fraction was stored at 4 °C for further analysis.

The homogeneity and subunit structure of the purified MGL from *A. flavipes* were checked by SDS‐PAGE^39^, with slight modifications^40,41^, compared to the protein ladder (Puregene, Cat. # PG‐PMT2962 315‐10 kDa).

### MGL activity and protein concentration

The MGL activity was determined by DTNB assay^42^, with slight modifications^17–20,24^. Briefly, the reaction mixture contains 40 mM L-methionine in Tris–HCl buffer (pH 7.8), 10 μM MPLP, 20 μl DTNB reagent and enzyme preparation in 1 ml total volume. The mixture was incubated for 15 min at 37 °C, and the developed color was measured at λ_412_ nm. Enzyme and substrate blanks were prepared. One unit of MGL activity was expressed by the amount of enzyme which releases 1 μmol of methanethiol per min under standard conditions. The protein content of enzyme was assessed by Folin’s reagent^43^ using bovine serum albumin as standard.

### Conjugation of *A. flavipes* MGL with β-cyclodextrin

The purified *A. flavipes* MGL was conjugated with the β-cyclodextrin^27^. Breifly, the β-cyclodextrin (50 mg) was activated by 5 mL Tris–HCl buffer (pH 7.0, 10 mM) for 1 h at room temperature, with stirring to get a uniformly mixed solution. Different molar ratios of the purified MGL (10, 30 and 60 μM) in Tris–HCl buffer (pH 7.0, 10 mM) was mixed with the activated β-cyclodextrin (10 mM), with gentle stirring for 12 h at 4 °C. The β-cyclodextrin-MGL (CD-MGL) conjugates were concentrated with 10 kDa centrifugal membrane at 1000 rpm for 1 min, to remove the extra unbounded cyclodextrin. The enzyme preparations were stored at 4 °C till use. The activity of the free and conjugated enzyme (CD-MGL) was determined as described above. The immobilization yield was determined by the activity of the CD-MGL to the free MGL × 100.

### FTIR analysis and modification of MGL surface reactive amino groups

The FTIR analyses of the native MGL and CD-MGL conjugates were conducted at wavelength of 400–4000 cm^−1^ using FTIR spectrometer model (JASCO, FT/IR-4100 type A).

The ratio of surface reactive ε-amino groups of lysine residues of MGL was assessed by fluorescamine assay^44^. The enzymes preparations were excited at *λ*_390_ nm, and the emitted fluorescence was scanned from *λ*_350–750_ nm, normalizing to Tris–HCl and β-cyclodextrin as blank baseline. The emitted fluorescence was expressed as arbitrary units.

The surface reactive amino groups of the MGL preparations were assessed by Ninhydrin reagent ^45^. The enzymes (10 μg/mL) were boiled with Ninhydrin for 5 min, and the percentage of the surface reactive amino groups was assessed by the absorbance at *λ*_575_ nm of the CD-MGL to the free enzyme.

### Proteolytic and chemical active sites mapping of the free and CD-MGL conjugates in vitro

The in vitro structural stability of the native MGL and CD-MGL conjugates in response to proteinase K and trypsin were assessed^17,21,23^. The enzymes preparations (20 μg/ml) were incubated with Trypsin and Proteinase K (10 μmol/min/mg) at 37 °C for 1 h, then PMSF was added to stop the proteolytic activity, and the remaining enzymatic activities were assessed by the standard assays as described above. The active site mapping of enzymes with the amino acids suicide analogues were used frequently^17,21,23^ by pre-incubating the enzymes with the amino acids suicide analogues namely guanidine thiocyanate, hydroxylamine, dithio-bis-(2-nitrobenzoic acid) (DTNB), 3-Methyl-2-benzothiazolinone hydrazone (MBTH) and PMSF (1 mM final Conc.) at 4 °C for 2 h, then measuring the residual enzyme activity as described above.

### Biochemical properties of free and dextran conjugated MGL

The optimum reaction temperature for the activity of free and CD-MGL conjugates was assessed by incubating the reaction at 4, 10, 20, 30, 37, 40, and 50 °C, then measuring the enzymatic activity as described above. The thermal stability of enzymes was evaluated by pre-incubating the enzyme preparations at the same concentration without substrate at 4, 15, 30, 37 and 50 °C for different time points, then measuring the residual enzymatic activities by the standard assay. The kinetic parameters; half-life time (*T*_*1/2*_), thermal inactivation rate (*K*_*r*_) and melting temperature (*T*_*m*_) were determined^17,20,21,23,46^. As well as, the impact of reaction pH on the activity of free and CD-MGL conjugates was assessed by adjusting the reaction at different pHs (4 to 8.5), then measuring the enzymatic activities by the standard assay. The pH stability of the enzyme preparations was evaluated by pre-incubating the enzymes without substrate at different pHs (5.0–8.0) using 50 mM citrate phosphate (pH 3.0–5.0), potassium phosphate (pH 5.6–7.8) and phosphate (pH 8.0–10.0) buffers. The putative precipitating pH of the free and CD-MGL was determined by incubating the enzymes without substrates at pHs 3.0–10.0 for 12 h at 4 °C, centrifugation at 10,000 rpm for 10 min, and the precipitated protein was measured^43^. The putative isoelectric focusing (*pI*) was expressed by the pH at which maximum protein was precipitated.

### Kinetic properties of the free and CD-MGL conjugates

The catalytic affinity of the free and CD-MGL conjugates for various amino acids; L-methionine, DL-homocysteine, L-cystine, L-cysteine, L-lysine, L-asparagine, L-tyrosine, L-glycine, L-phenylalanine, and L-alanine were evaluated, based on their deaminating and demethiolating activities^17–20,26^. The kinetic and catalytic parameters; Michalis-Menten constant (*K*_M_), maximum velocity (*V*_max_), turnover number (*K*_cat_) and catalytic efficiency (*K*_cat_*/K*_M_) of the selected substrates were assessed from the Lineweaver–Burk plot model. The MGL activity was determined based on amount of released thiols and ammonia.

### Anticancer activity of the free and CD-MGL in vitro and in vivo

The activity of free and CD-MGL conjugates towards the breast carcinoma (MCF7), colon cancer cells (HCT116), and Oral Epithelial cells (OEC), was evaluated by the MTT assay^47^. The 96-well plate was seeded with 10 × 10^3^ cells per well, incubated for 12 h at 37 °C, then amended with different enzymes concentrations, and then the plates were re-incubated for 48 h at the same conditions. The MTT reagent was added, incubated for 2 h, and the developed formazan complex was dissolved in 100 μl DMSO, and the developed colored complex was measured at *λ*_570_ nm. The viability of cells was determined by the A_570_ nm of sample compared to control × 100. The IC_50_ value was expressed by the enzyme activity reducing 50% of the initial number of cells compared to phosphate buffered saline as control, according to the formula;

$$\begin{aligned} {\text{Cell viability }}\left( \% \right) & = {\text{Absorbance at }}\lambda _{{570}} {\text{of the enzyme}} \\ & \quad - {\text{treated cells}}/{\text{ Absorbance at }}\lambda _{{570}} {\text{of control cells}} \times 100 \\ \end{aligned}$$

The cytotoxic properties of the free and CD-MGL conjugates were assessed in BABL/c albino female mice (20 g of 25 days old) according to the guidelines of the Institutional Animal Care and Use Committee of the Faculty of Medicine, Zagazig University, and NIH guidelines under protocol 15-08-263, following the institutional guidelines for the human treatment of animals, the Principles of Laboratory Animal Care (National Institutes of Health, Bethesda, MD, USA). The mice were acclimatized for 5 days, prior to enzymes injection, grouped into five groups: 1- Negative control, mice without Ehrlich Ascites Carcinoma (EAC), 2- Positive control, mice were intraperitoneal injected with 2.5 × 10^6^ of EAC cells, incubated for 5 days till the tumor size reached to 50 mm^3^. 3- Treated group with the free MGL, the eight days post-inoculated mice of EAC were injected intravenously with single dose of free MGL. 4- Treated group with CD-MGL, the eight days post-inoculated mice of EAC were injected with single dose of CD-MGL. 5-Negative control group treated with the single dose of the free MGL and CD-MGL, separately. Each group has five animals, and the data were represented by the means and standard deviations. The enzymes were sterilized by 0.45 μm membrane filter prior to injection. The mice were anesthetized with urethane, sacrificed by cervical dislocation^35^. The EAC were collected from the mice, rinsed with saline, and subjected to various histopathological analyses.

The activities of the free MGL and CD-MGL were measured in the blood samples intervally collected after 0.5, 2.0, 6, 12 and 24 h of the initial enzymes dosing of the 5th group, as mentioned above^42^. Briefly, the reaction mixture contains 40 mM L-methionine in Tris–HCl buffer (pH 7.8), 10 μM MPLP, 20 μl DTNB reagent and 100 μl of blood plasma in 1 ml total volume, incubated for 15 min at 37 °C, followed by 10% TCA. The developed color was measured at *λ*_412_ nm, normalized to blank of plasma-free enzymes^20^. The biological half-life time of the free and CD-MGL was calculated from the linear equation of their activity in vivo^42^.

### Molecular docking analysis

#### Biological data, and 3D structure and molecular docking analysis

The crystal structure of MGL from *Pseudomonas putida* was used from the EMBL-EBI database^48^. The domain was derived from the X-ray crystallographic structure of *P. putida* MGL with a 1.8 Å resolution (PDB ID: 3vk3) and 398 amino acid sequence (UniProtKB ID: P13254) (https://www.uniprot.org/uniprotkb/P13254/entry). The MGL of *A. flavipes* was conjugated by host–guest chemistry to β-cyclodextrin. Energy minimization was conducted using open-source molecular builder, visualization tool (http://avogadro.cc/), ligand preparation by Open Babel (Version 2.3.1) following by analysis pipeline^49^ for preparing small-molecule libraries.

The enzyme consists of four molecular subunits (A, B, C and D) as reveled from the Protein databank^48^, the subunit A was used for the modeling analysis with the Discovery Studio software (version 2019). Random setting was used for orientating the torsions of β-cyclodextrin, with the Auto Dock Vina software package (Version 2.0) for grid box generation. The enzyme was docked with β-cyclodextrin, by the Auto Dock to rank the results included binding energy implicated in chemical interactions, hydrogen bonds, and hydrophobic regions. LIGPLOT was utilized for analyzing the hydrophobic and H -bond interactions between the ligands and domain complexes.

#### Statistical analysis

All the experiments were excused in three biological replicates, and the results were represented by the mean ± STDV. The results were analyzed by one-way ANOVA.

## Results

### Screening for the optimum nutritional requirements for maximum MGL production of *A. flavipes* by Response Surface Methodology

**Fig. 1:**
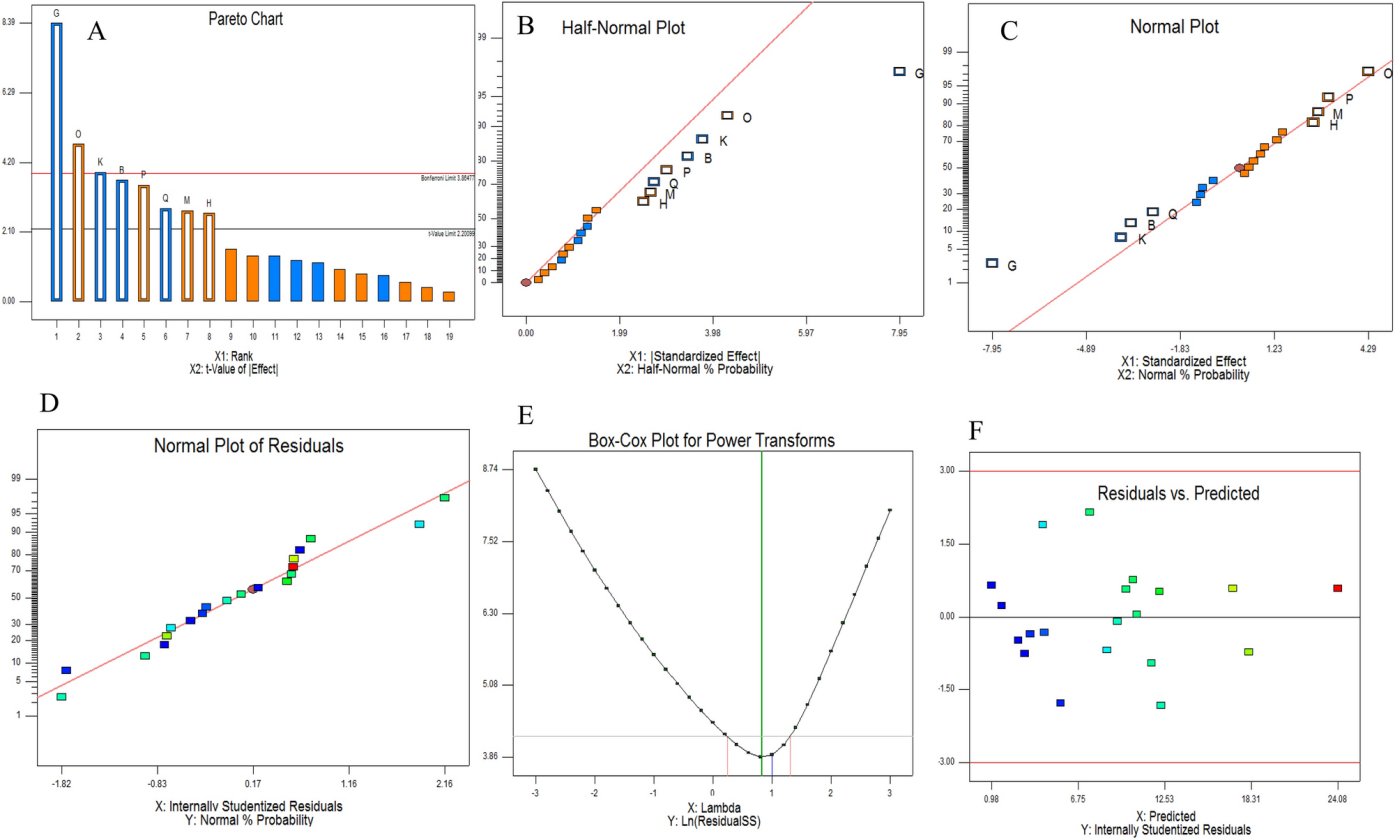
Response Surface Methodology nutritional optimization of MGL production from *A. flavipes* with the Plackett–Burman experimental design. (**A**), Pareto chart illustrates the order of significance of each variable. B, Half-normal (**B**) and normal probability plots (**C**) of the variables for MGL production as determined by the first-order polynomial equation. (**D**), Normal plot of the residuals. (**E**), Box-Cox power transform of the residuals. (**F**), Plot of the residuals and predicted MGL productivity by *A. flavipes*.

The influence of nineteen independent variables of different carbon, and nitrogen sources, trace elements and vitamins on MGL productivity by *A. flavipes* was studies by the Plackett–Burman Design (Table S1). After cultural incubation at the desired conditions, the intracellular crude proteins were extracted, and the enzyme activity and concentration were determined. From the Plackett–Burman design matrix, the predicted and actual values of MGL productivity by *A. flavipes* was summarized in Table 1, showing a significant independent variables effect on enzyme productivity. A high fluctuation on the predicated and actual activity of MGL was observed, that ranged from 2.0 to 13.0 μmol/mg/min, reflects the significance effect of the tested variables. The highest actual and predicted productivity of MGL by *A. flavipes* was 13 and 12 μmol/mg/min, respectively, at the run number 18. The maximum MGL productivity by *A. flavipes* (13 μmol/mg/min) was reported at run # 18, followed by run #10, and run # 17. So, the highest *A. flavipes* MGL productivity was detected by growing the fungus on medium with L-methionine ( − 1), L-glycine ( − 1), Valine ( − 1), L-asparagine ( − 1), L-glutamine, L-lysine, D-glucose, fructose (+ 1), xylose (+ 1), and vitamin B6 ( − 1) at pH 6.0, with 6 days incubation at 30 °C. The significance of the tested variables on MGL productivity was revealed from Pareto Chart, plots of probability of independent variables, and plots of actual and predicted enzyme activity (Fig. 1). The significance of variables coefficients as revealed from the *p*-value and student’s t-test was summarized in Table S2. The points of residuals were arranged near the diagonal line, revealing the independent normal distribution of variables, ensuring the good- fitting of the expected and actual yield of MGL by *A. flavipes.* The constructed model was highly significant as revealed from the values of F-test 7.4 as reveled from the Plackett–Burman design (Table S2). The first-ordered polynomial equation of MGL production by *A. flavipes* was represented as follows:

**Table 1:** Plackett–Burman experimental design of the nineteen independent variables on the activity of MGL from *Aspergillus flavipes*. The “ − 1” and “ + 1” signs corresponding to the minimum and maximum values of the input parameter range.

	X1	X2	X3	X4	X5	X6	X7	X8	X9	X10	X11	X12	X13	X14	X15	X16	X17	X18	X19	Actual	Predicted	Residuals
1	2	2	4	4	4	4	2	8	2	4	2	0.1	0.1	0.1	0.6	0.6	6	6	100	5	2	0.133
2	4	2	2	4	4	4	10	2	8	1	8	0.1	0.1	0.1	0.2	0.6	8	10	150	2	4.8	− 96
3	2	4	4	4	4	2	10	2	8	1	2	0.1	0.1	0.4	0.6	0.2	8	6	100	5	4.6	3.325
4	4	4	2	4	4	2	2	8	8	4	8	0.1	0.4	0.1	0.6	0.2	6	10	100	5	4.94	0.893
5	2	2	4	4	2	4	10	2	2	4	8	0.4	0.4	0.1	0.6	0.2	8	6	100	1.4	2.6	− 2.8
6	4	4	4	2	4	2	10	2	2	1	2	0.4	0.4	0.1	0.6	0.6	6	6	150	1	2.2	− 1.2
7	4	2	2	2	2	4	10	2	8	4	2	0.1	0.4	0.4	0.6	0.6	6	10	100	1.4	1.5	− 0.5
8	2	4	2	4	2	2	2	2	8	4	2	0.4	0.4	0.1	0.2	0.6	8	10	150	3.9	2.4	2.967
9	2	4	4	2	2	4	10	8	8	1	8	0.1	0.4	0.1	0.2	0.2	6	10	150	1	2.7	− 0.7
10	4	4	2	4	2	4	2	2	2	1	8	0.4	0.1	0.4	0.6	0.2	6	10	150	8	9.2	− 1.2
11	4	2	4	4	2	2	10	8	8	4	2	0.4	0.1	0.4	0.2	0.2	6	6	150	4	4.3	− 0.08
12	2	2	2	4	4	2	10	8	2	1	8	0.4	0.4	0.4	0.2	0.6	6	10	100	6	5.6	1.017
13	4	4	2	2	4	4	10	8	2	4	2	0.4	0.1	0.1	0.2	0.2	8	10	100	2	1.7	0.3
14	4	4	4	4	2	4	2	8	2	1	2	0.1	0.4	0.4	0.2	0.6	8	6	100	4.4	6.2	− 2.78
15	2	2	2	2	2	2	2	2	2	1	2	0.1	0.1	0.1	0.2	0.2	6	6	100	5.1	6.6	− 1.45
16	4	2	4	2	4	2	2	2	2	4	8	0.1	0.4	0.4	0.2	0.2	8	10	150	6	7.2	0.8
17	4	2	4	2	2	2	2	8	8	1	8	0.4	0.1	0.1	0.6	0.6	8	10	100	9	8	1
18	2	2	2	2	4	4	2	8	8	1	2	0.4	0.4	0.4	0.6	0.2	8	6	150	13	12	1
19	2	4	4	2	4	4	2	2	8	4	8	0.4	0.1	0.4	0.2	0.6	6	6	100	4.6	5.7	− 1.1
20	2	4	2	2	2	2	10	8	2	4	8	0.1	0.1	0.4	0.6	0.6	8	6	150	2	2.6	− 0.6

MGL activity = 15.0439–1.7187 * glycine − 0.9942 *glucose + 0.4162* fructose − 1.2519 *lactose + 8.8472 * MgCl_2_ + 14.2972 * CaCl_2_ + 7.4812 * vitamin B6 − 6.7979 *vitamin B_12_.

Thus, the productivity of MGL by *A. flavipes* was increased by about two folds (13.2 μmol/mg/min) compared to the control (6.5 μmol/mg/ min) in response to Plackett–Burman design. .

### Purification and molecular subunit structure of MGL from *A. flavipes*

**Fig. 2:**
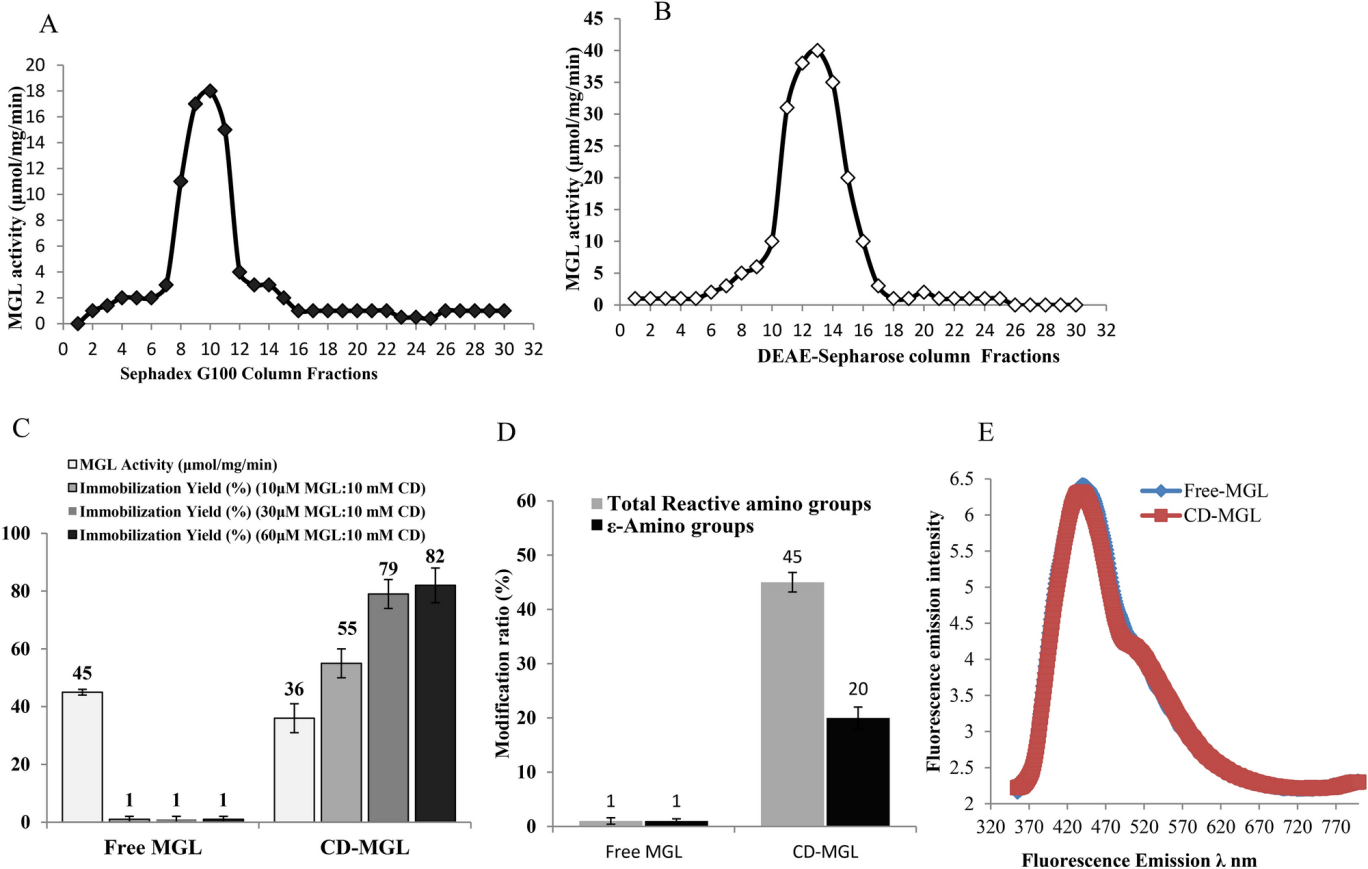
Purification, molecular subunit structure and conjugation with cyclodextrin of MGL from Aspergillus flavipes. The intracellular crude proteins was extracted, acetone precipitated, and the enzyme was fractionated with gel-filtration column (**A**), and Ion-exchange chromatography column (**B**). The purified MGL was conjugated with cyclodextrin, and the activity and immobilization yield of CD-MGL was measured (**C**). (**D**), The total reactive surface amino groups. (**E**), The fluorescence emission of the free MGL and CD-MGL at 300 nm excitation.

*Aspergillus flavipes* was grown on the recovered media from the Plackett–Burman design, incubated at 30 °C for 7 days. After incubation, the fungal pellets (~ 100 gm) were collected, washed and pulverized in liquid nitrogen, dispensed in 100 mL Tris–HCl buffer (pH 7.2, 10 mM) and the intracellular crude proteins were extracted, concentrated by dialysis membrane of 20 kDa cut-off, till minimal volume, then further concentrated by 30 kDa Centrifugal membrane. The overall purification profile of MGL from *A. flavipes* was summarized in Table 2. The enzyme was purified by Gel-filtration chromatography with Sephadex G100 column, the activity of eluted fractions was assessed (Fig. 2A). Upon gel-filtration, the activity of MGL was increased by 1.2 folds (15 μmol/mg/min) with 87.5% yield with Sephadex G100 column. The active fractions were gathered and purified by ion-exchange chromatography with DEAE-Sepharose column, the fractions were eluted by 100 mM NaCl, and the enzyme activity was assessed (Fig. 2B). The enzyme was purified by about 3.2 folds (38 μmol/mg/min) with yield 82.3% compared to the crude enzyme. Overall, with the gel-filtration and ion-exchange chromatographic approaches, the enzyme activity was increased to 38.1 μmol/mg/min, with about 3.2 folds of purification increments and 82.3% yield, compared to the crude enzyme. The molecular subunit structure of the purified MGL from *A. flavipes* was determined by denaturing-PAGE. From the SDS-PAGE profile (Fig. S1), a single homogenous band of about 48.0 kDa was appeared after the last purification step, revealing the efficiency of the designed protocol for purification of intracellular MGL from *A. flavipes*.

**Table 2:** Overall purification profile of L-methionine γ-lyase from *Aspergillus flavipes.*

	Activity (μmol/min)	Protein (mg/ml)	Specific activity (μmol/mg/min)	Yield (%)	Purification fold
Crude-MGL	60	5	12	100	1
Acetone precipitation	57.2	4.4	13	95.3	1.08
Sephadex G100	52.5	3.5	15	87.5	1.25
DEAE-Sepharose	49.4	1.3	38.1	82.3	3.2

### Conjugation of *A. flavipes* MGL with β-cyclodextrin, modification of the surface reactive amino groups

The purified *A. flavipes* MGL was conjugated with β-cyclodextrin at different molar ratios of MGL/β-cyclodextrin (μM/mM) (10:10, 30:10, and 60:10). The unbounded cyclodextrin residues without MGL were removed by ultracentrifugal membrane 10 kDa at 1000 rpm for 5 min. The activity of the MGL conjugated with β-cyclodextrin (CD-MGL) was determined by the standard assay, and the yield of MGL immobilization of was calculated. From the results (Fig. 2C), the immobilization yield of MGL with β-cyclodextrin was increased gradually with the enzyme concentration, till the maximum value (80%) at molar ratio 30:10 and 60:10 of MGL/ β-cyclodextrin, with the activity of the CD-MGL 36–38 μmol/mg/min, compared to the free MGL (45 μmol/mg/min). The slight reduction on the activity of CD-MGL might be due to the slight shielding masking of surface enzyme catalytic sites.

The degree of conjugation of MGL with β-cyclodextrin was assessed based on the frequency of the total surface reactive primary, secondary and ε-amino groups of MGL as described in Materials and Methods. From the results, the reactivity of MGL surface amino groups was reduced by about 45% upon β-cyclodextrin conjugation, ensuring the occupation of about 55% of the enzyme surface amino groups via interaction with β-cyclodextrin (Fig. 2D). The reactivity of ε-amino groups of the MGL surface lysine residues was assessed by the fluorescamine reagent at 300 nm excitation wavelength. Interestingly, the emitted fluorescence of both free and CD-MGL have the same pattern of fluorescence mission, ensuring the lack of interaction with the surface ε-amino groups of MGL upon β-cyclodextrin conjugation (Fig. 2E).

**Fig. 3:**
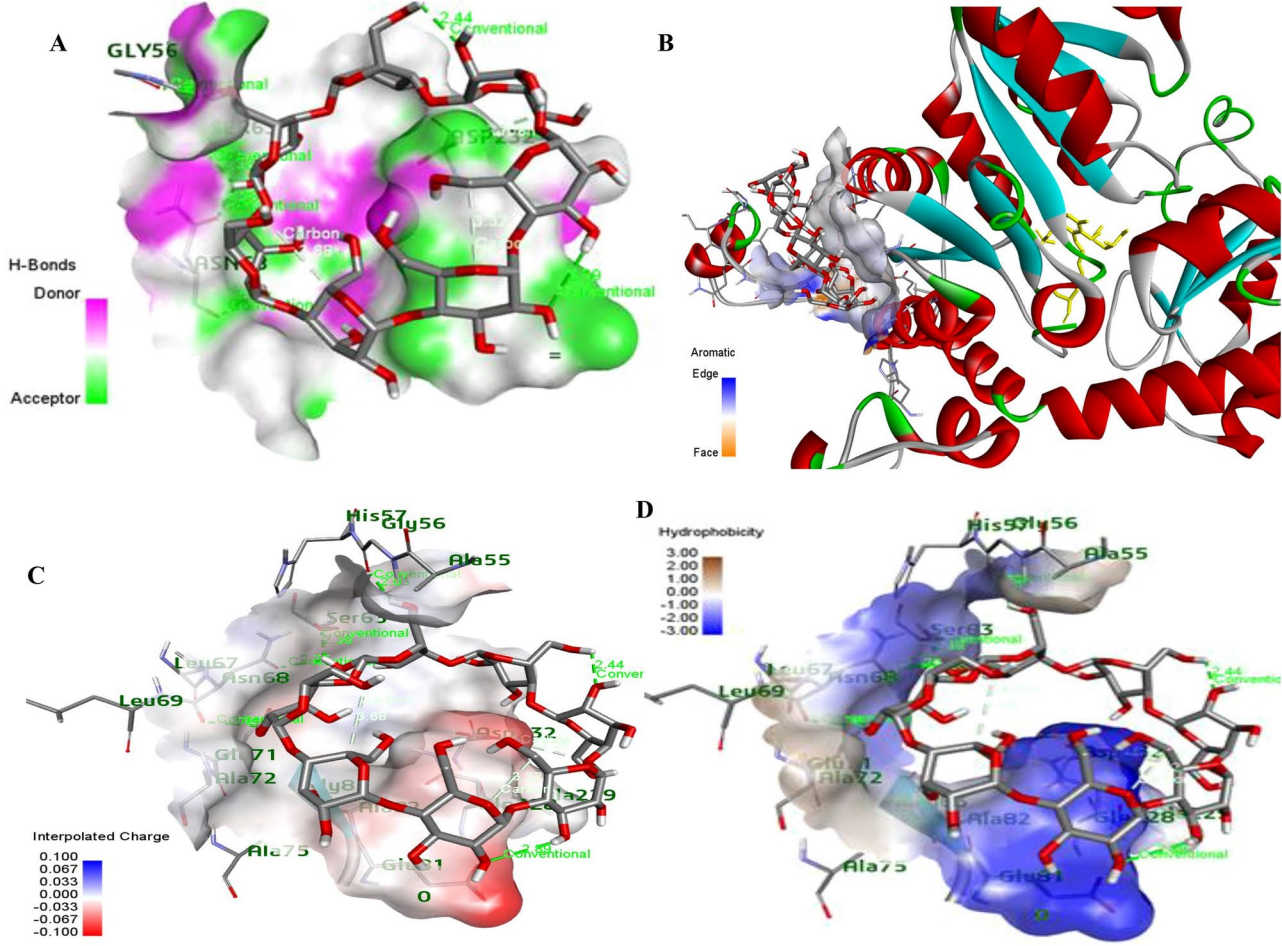
The molecular modeling analysis of MGL with β-cyclodextrin was conducted to assess the possible molecular interactions of enzyme with cyclodextrin. The H-bonds (**A**), Aromatic edges (**B**), Interpolated charges (**C**) and hydrophobicity of the surface residues (**D**) was resolved from the docking analysis.

The molecular modeling analysis of MGL with β-cyclodextrin was conducted to assess the possible molecular interactions of enzyme and cyclodextrin. From the modeling analysis (Fig. 3), the MGL binds with the cyclodextrin with various non-covalent, and hydrogen bonding interactions. These interactions were occurred between the surface aromatic amino acids of MGL mainly tyrosine, phenylalanine, tryptophan, in addition to serine, asparagine, alanine, and glutamic acid and hydroxyl groups of the cyclodextrins. The chemical interactions of the MGL with cyclodextrins via the interpolated charges, hydrophobicity and hydrogen bonding were shown in Fig. 3.

### FTIR analysis of the free and CD-MGL conjugates

**Fig. 4:**
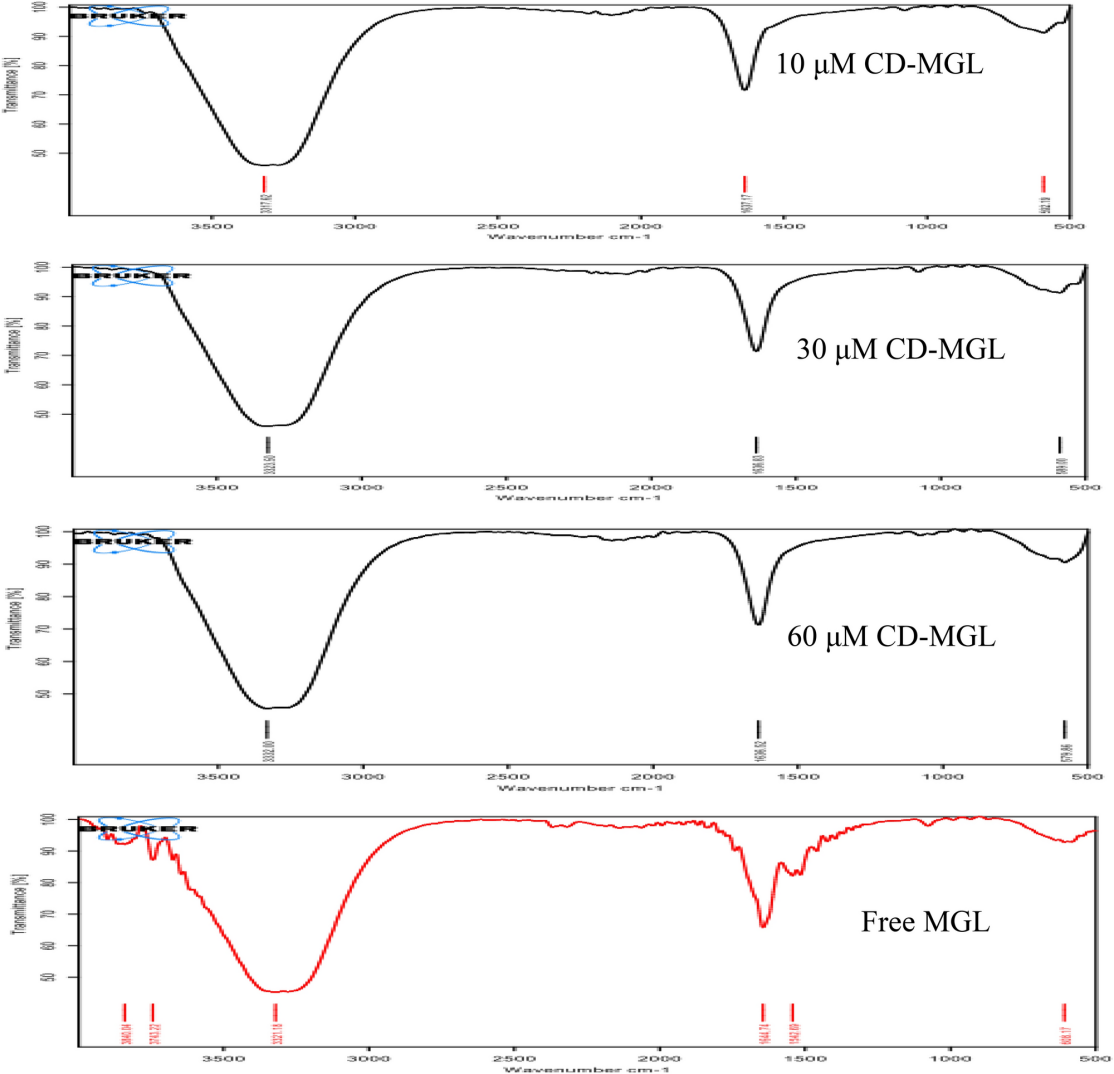
FTIR spectroscopic analysis of the free and CD-MGL conjugates at different molar ratios of the MGL (10–60 μM with 10 mM cyclodextrin.

The chemical interactions of the MGL with β-cyclodextrin were evaluated by the FTIR spectroscopy, using different molar ratios of the purified MGL (10, 30, and 60 μM) with 10 mM β-cyclodextrin. From the FTIR spectra (Fig. 4), the enzyme has a broad band characteristic peak spectrum centered at ~ 3400 cm^−1^ that arises from the stretching of hydroxyl groups, in addition to band at ~ 2900 cm^-1^ that attributed to CH_2_ groups. Also, the enzyme had a peak at 1620 cm^−1^ and 1418 cm^−1^ that refers to the COO^−^ asymmetric and symmetric stretching. The band at 1631 cm^−1^ refers to OH bending vibration of adsorbed water molecules, 1035 cm^−1^ refers to C–O stretching, 613 cm^−1^ refers to hydrogen bonds on the hetero-aromatic nucleus, and at 900–600 cm^−1^ refers to C–H stretching vibrations. A slight shifts on the surface functional groups of MGL upon β-cyclodextrin conjugation has been observed, reveling the interaction of the surface hydroxyl, and carboxyl groups with the glucose units of β-cyclodextrin. For the CD-MGL, a band centered at 3435 cm^−1^ was appeared refers to the stretching vibration of the hydroxyl groups, while the band at 1630 cm^−1^ reveals the bending vibration of O–H bonds of adsorbed water molecules on the cyclodextrin surface. The emerged band of CD-MGL at 2164 cm^−1^ referred to CO_2_ group. The observed peak at 1305 cm^−1^ refers to the asymmetrical stretching C–O group. The slight stretching on the hydroxyl groups “Van der Waals forces” at 3320, 3334 and 3330 cm^−1^ for the MGL upon cyclodextrin conjugation.

### In vitro proteolysis of free and CD-MGL conjugates

The effect of conjugation of MGL with β-cyclodextrin on the accessibility of surface proteolytic recognition sites of MGL was determined by incubating the native and CD-MGL at the same concentrations, 50 μg/mL) with 10 U proteinase K and trypsin at 37 °C, and then measuring their residual activity by the standard assay. From the results (Fig. S2A), the residual activity of the native and CD-MGL conjugate was 10% and 60%, respectively, after 30 min of proteolysis with proteinase K. The activity of the free and CD-MGL due to trypsin digestion after 30 min were 34 and 78%, respectively (Fig. S2B). The higher residual activity of the free MGL in response to trypsin than proteinase K, reveals the higher frequency of recognition sites for proteinase K than trypsin one. The higher resistance of CD-MGL to trypsin digestion ensures the masking effect of cyclodextrin to the surface proteolytic recognition sites of MGL. The number of recognition sites for proteinase K was 220 sites as calculated by the peptide cutter (ExPASY, Bioinformatics Resource Portal). While, the predicted recognitions sites for trypsin was about 24 sites, that mostly for the consecutive amino acids lysine-valine, arginine-glutamic, arginine-leucine, lysine-leucine, lysine-phenylalanine, lysine-histidine, lysine-tyrosine, and lysine-leucine. The proteolytic resistance of MGL upon cyclodextrin conjugation might be due to the blocking of these reactive groups by interaction with cyclodextrin.

The impact of various amino acids suicides analogues on the catalytic structure of the free and CD-MGL was assessed by pre-incubating with hydroxylamine, iodoacetamide, guanidine thiocyanate, DTNB, MBTH, H_2_O_2_, PMSF and HgCl_2_, then measuring their residual activities. Conjugation of MGL with cyclodextrin dramatically stabilizes enzyme catalytic structure, as revealed from the relative activity (Fig. S2). The free MGL lost about 60–70% of its initial activity with hydroxylamine, PMSF, DTNB, MBTH, and guanidine thiocyanate, in contrary, the CD-MGL displayed a strong stability to these inhibitors, that approximated by < 10% of their initial activity. The dramatic stability of the CD-MGL due to these amino acids analogues reveals the stabilizing effect of cyclodextrin to the enzyme that could be due to the shielding/ masking of the surface cysteine residues, glutamine, asparagine, and lysine residues. Thus, cyclodextrin conjugation of the MGL could reduce the accessibility of the enzyme surface amino acids especially cysteine, glutamine, asparagine, lysine to the tested chemical inhibitors^17,21^. The DTNB and MBTH compounds mainly bind with the surface cysteinyl and primary amino groups containing amino acids “glutamine, asparagine”, and the strong relative activity of CD-MGL to these compounds could be due to the hiding of these amino acids from the accessibility for the reactivity of these compounds.

### Biochemical properties of the native and CD-MGL conjugates

The properties of the free and CD-MGL “reaction temperature, thermal stability, reaction pH, and pH stability were estimated. The standard reaction mixture of the free and CD-MGL was incubated at 4, 10, 15, 20, 25, 30, 35, 40 and 50 °C for 15 min, then the enzymatic activity were measured. Obviously, from the profile of reaction temperature, the free and CD-MGL conjugates have the same activity pattern to reaction temperature. The maximum activity of free and CD-MGL conjugates (42.5 μmol/mg/min) were obtained at 37–40 °C (Fig. S3A). The similar activity of the free and CD-MGL in response to the reaction temperature, ensures the lacks of interaction of cyclodextrin with the catalytic sites of MGL, and subsequently binding with substrate and catalysis process.

The catalytic behavior of the free and CD-MGL to the different pHs was evaluated by adjusting the reaction mixtures at pH range 3–10, with different buffers, and the enzymatic activities were measured by the standard assay. The free enzyme and CD-MGL conjugates exhibited the same maximum activity at pH range 7.0–80 in potassium phosphate buffer (10 mM), with a noticeably significant reduction to the activity of both enzymes at highly acidic pHs (3.0–4.0), compared to the slight effect at alkaline pHs (8.5–10) (Fig. S3B), The same catalytic response of the free and CD-MGL, authenticates the absence of negative interactions of cyclodextrin with the surface charged residues of the enzyme, cyclodextrin conjugation has no effect on the net charge of the enzyme. The pH stability of the free and CD-MGL conjugates was assessed by pre-incubating the enzymes at different pHs (6.0–9.0), for 2 h, then determining their residual activities as described above. The maximum catalytic stability of the free and CD-MGL was observed at pH range 6.6–8.0, with a dramatic reduction to their activities at pH 5.0 (Fig. S3C). Remarkably, the free and CD-MGL have the same pattern of pH stability, suggesting the lack of cyclodextrin interactions with the charged catalytic residues of enzyme. The putative isoelectric points of the free and CD-MGL conjugates were explored from their pH precipitation profiles^21^, at the same enzymes concentrations. Practically, a noticeable shift on the putative pH precipitation for the MGL was observed upon conjugation with β-cyclodextrin, in addition to the amount of precipitated proteins “solubility of enzymes”. The maximum pH precipitation (*p*I) of the free MGL and CD-MGL was 6.0 and 5.0, respectively, confirming the acquired higher solubility, resistance to denaturation of MGL upon cyclodextrin conjugation. The precipitation of the enzymes at the corresponding *pI,* are usually due to the reorientation of the aromatic amino acids “hydrophobic” (tyrosine, phenylalanine, tryptophan) from the core of enzyme molecule to the surface, causing protein precipitation^34,50^.

### Kinetics properties of free and CD-MGL conjugates

The affinity of free and CD-MGL conjugates towards various amino acids “L-methionine, DL-homocysteine, L-cysteine, L-cystine, L-lysine, L-asparagine, L-valine, L-tyrosine, L-glycine, L-phenylalanine, L-alanine and glutamine” were assessed. The enzymatic activities were determined based on the released ammonia and methanethiol. From the substrate specificity (Table S3), the free and CD-MGL had the highest affinity for L-methionine followed by DL-homocysteine based on their demethiolating activity. The relative activity of the free and CD-MGL for DL-homocysteine 94–96% and for L-cysteine were 15–18%, compared to L-methionine as standard substrate. A mild-to-nil activity of the free and CD-MGL towards the other tested amino acids, based on the deaminating activity. The same pattern of catalytic affinity of the free and modified enzyme towards the sulfur-containing amino acids as substrates ensures the lack of chemical interactions of cyclodextrin with the enzyme catalytic domains and enzyme tertiary structure. The kinetic properties of the free and CD-MGL for L-methionine, DL-homocysteine and L-cysteine (10–100 μM), as substrates was assessed. The overall kinetic properties of the enzymes were summarized in Table 3. Practically, the kinetics properties of the free MGL for L-methionine as standard substrate were greatly improved upon modification with cyclodextrin. The catalytic affinity (*K*_M_) of the free and CD-MGL conjugates for L-methionine was 7.2 mM and 3.1 mM, respectively, thus, the affinity of MGL for L-methionine was increased by two folds, upon cyclodextrin conjugation. As well as, the maximum velocity (*V*_*max*_) of the native enzyme (32.1 μmol/mg/min) was increased by two folds (60.1 μmol/mg/min) upon cyclodextrin conjugation, ensuring the improved kinetic properties of CD-MGL compared to the native one. Additionally, the catalytic efficiency (*K*_cat_/*K*_M_) of the native enzyme (1.5 mM^−1^ s^−1^) was enhanced by ~ 1.4 folds upon cyclodextrin conjugation (2.5 mM^−1^ s^−1^). The slight fluctuation on catalytic affinity of MGL in response to cyclodextrin conjugation might be correlated to the re-orientation and re-shaping of the surface catalytic, and allosteric domains of the enzyme, that might be due to interactions of the surface amino acids with the hydroxyl functional groups of cyclodextrin.

**Table 3:** Kinetics of the free and CD-MGL for L-methionine, L-cysteine and DL-Homocysteine.

	Free-MGL				CD-MGL			
	*K*_*m*_ (mM)	*V*_*max*_ (μmol/mg/min)	*K*_*cat*_ (s^-1^)	*K*_*cat*_*/K*_*m*_ (mM^-1^ s^-1^)	*K*_*m*_ (mM)	*V*_*max*_ (μmol/mg/min)	*K*_*cat*_ (s^-1^)	*K*_*cat*_*/K*_*m*_ (mM^-1^ s^-1^)
L-Methionine	7.2	32.1	9.9	1.5	3.1	60.1	19.7	2.5
Homocysteine	12.2	15.5	4.1	0.9	12.1	20.3	6.1	1.3
L-Cysteine	9.5	5.6	1.2	0.6	9.2	5.2	1.1	0.8

**Fig. 5:**
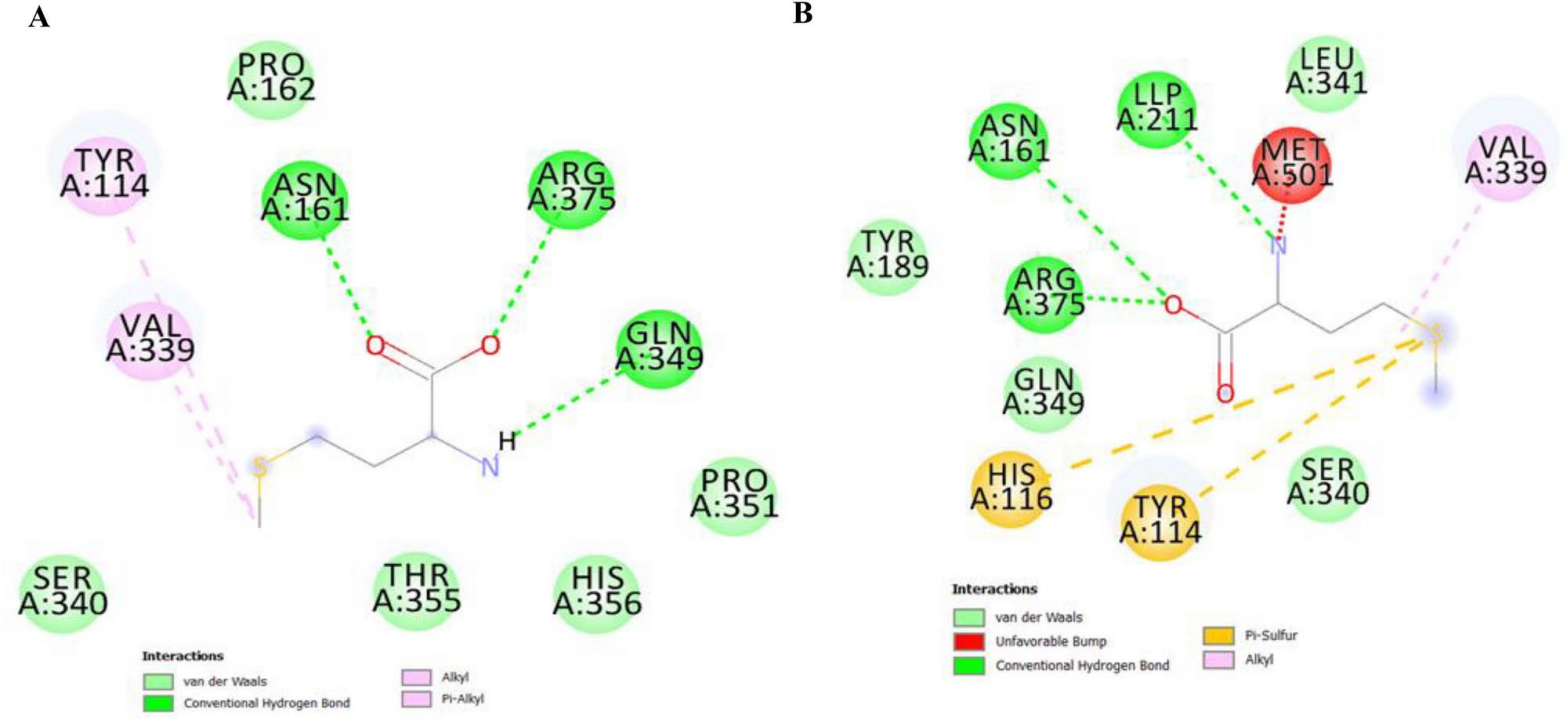
Molecular docking analyses of the free enzyme and CD-MGL with the L-methionine as L-methionine. A, Interactions of L-methionine with the free MGL (**A**), and their ionizability (**B**). Interactions of L-methionine with CD-MGL (**C**) and their ionizability (**D**).

The affinity of the MGL towards the tested substrate has been explored from the molecular docking analyses of the free enzyme and CD-MGL with the L-methionine as standard substrate. From the molecular docking analysis of the free MGL with the L-methionine (Fig. 5), L-methionine interacted with the different catalytic domains of MGL by about three hydrogen bonds and two hydrophobic bonds. The hydrogen bonds were reported between L-methionine and Gln 349, Arg373, ASN 161, while the hydrophobic bonds (Pi-alkyl bonds) were between Tyr 114 and Val339. However, with the docking analysis of L-methionine as substrate with CD-MGL, the substrate was interacted with the surface amino acids by seven bonds. Three hydrogen bonds were resolved between L-methionine with LLP211, ASN161, ARG375, two hydrophobic bonds for His116, Tyr114, and covalent bond with Met501. The higher interacting bonds of L-methionine with the surface amino acids residues of MGL upon cyclodextrin conjugation, could be correlated with the affinity of MGL to L-methionine as substrate. The development of covalent bonds between L-methionine as substrate and MET 501 of the MGL, could be the reason for the higher affinity of CD-MGL towards L-methionine, compared to the free MGL.

### Thermal conformational stability of the free and CD-MGL conjugates

The thermal conformational stability of the free and CD-MGL conjugates in response to thermal treatments at 4, 15, 30, 37 and 50 °C, for 30, 60, 120, and 250 min, without substrate, were assessed by the standard assay. From the thermal stability profile (Fig. S3), the catalytic stability of MGL was strongly increased upon conjugation with β-cyclodextrin, along the tested temperature and time points. The thermal kinetic parameters of the free and CD-MGL conjugates were summarized in Table 4. From the storage stability at 4 °C, the half-life time (*T*_*1/2*_) of the free and CD-MGL conjugates were 9.2 days and 16.2 days, respectively, ensuring the acquired conformational stability of enzyme upon cyclodextrin conjugation. By preserving the enzymes at 15 °C, the free and CD-MGL lost approximately 50% of their activities after 139.9 h and 166 h, respectively. The half-life times (*T*_*1/2*_) of the free and CD-MGL at 37 °C, were ~ 23.5 h and 53.2 h, respectively, i.e. by about 2.3 stabilization fold. Interestingly, upon conjugation with cyclodextrin the tertiary and catalytic structure of MGL were stabilized by ~ 1.7 to 2.3 folds along the tested thermal treatments. The thermal denaturation rate (*K*_*r*_) of the free MGL was dramatically increased with the higher thermal treatments, unlike to the corresponding values of the CD-MGL. The structural thermal stability of MGL was strongly increased upon conjugation with cyclodextrin, as revealed stabilizing factors (Table 4). At 4 °C, the thermal denaturation rate (Kr) of the free and CD-MGL conjugates was 30 × 10^–3^ min^-1^, and 14.6 × 10^–3^ min^-1^, respectively, by about 1.8 stabilization folds, ensuring the stabilizing effect of cyclodextrin on MGL conformational structure. While at 50 °C, the *K*_*r*_ values were 62.3 × 10^–3^ min^-1^ and 35.8 × 10^–3^ min^-1^ for the free and CD-MGL conjugates, with an obvious stabilizing effect by cyclodextrin by ~ 2 folds. As well as, the putative melting temperature (*T*_*m*_) of the free and CD-MGL were 62 °C and 140 °C, respectively, with a noticeable ~ 2 folds increase upon cyclodextrin conjugation. Overall, upon cyclodextrin conjugation the stability and catalytic efficiency of MGL was increased by ~ 2 folds, along the experimented thermal treatments.

**Table 4:** Thermal kinetic parameters of the free and Cyclodextrin MGL conjugates.

	Free-MGL			CD-MGL			
(°C)	T_1/2_ (h)	*Kr* x (10^–3^) min^−1^	*T*_*m*_ (°C)	T1/2 (h)	*Kr* x × 10–3 min^-1^	*T*_*m*_ (°C)	Stabilizing Factor
4	220.87	30	62	387.34	14.69	140	1.753662
15	139.97	40.2		166	20		1.185895
30	57.323	58.2		101.37	25.98		1.768518
37	23.5309	57.9		53.15	30.265		2.259053
50	14.9237	62.37		31.42	35.822		2.105529

*Half-life time (T1/2) was expressed by time which the enzyme retains 50% of its initial activity by preheating without substrate at each temperature degree.

**Thermal denaturation rate (Kr) was expressed by the logarithmic decreasing of enzyme activity with the time at each temperature. ln (At/A0) = —Kr, where A0 and At are the specific activity of the enzyme at zero and t time. It described by the first ordered kinetic model.

### Anticancer activity of the free and CD-MGL conjugates in vitro

**Fig. 6:**
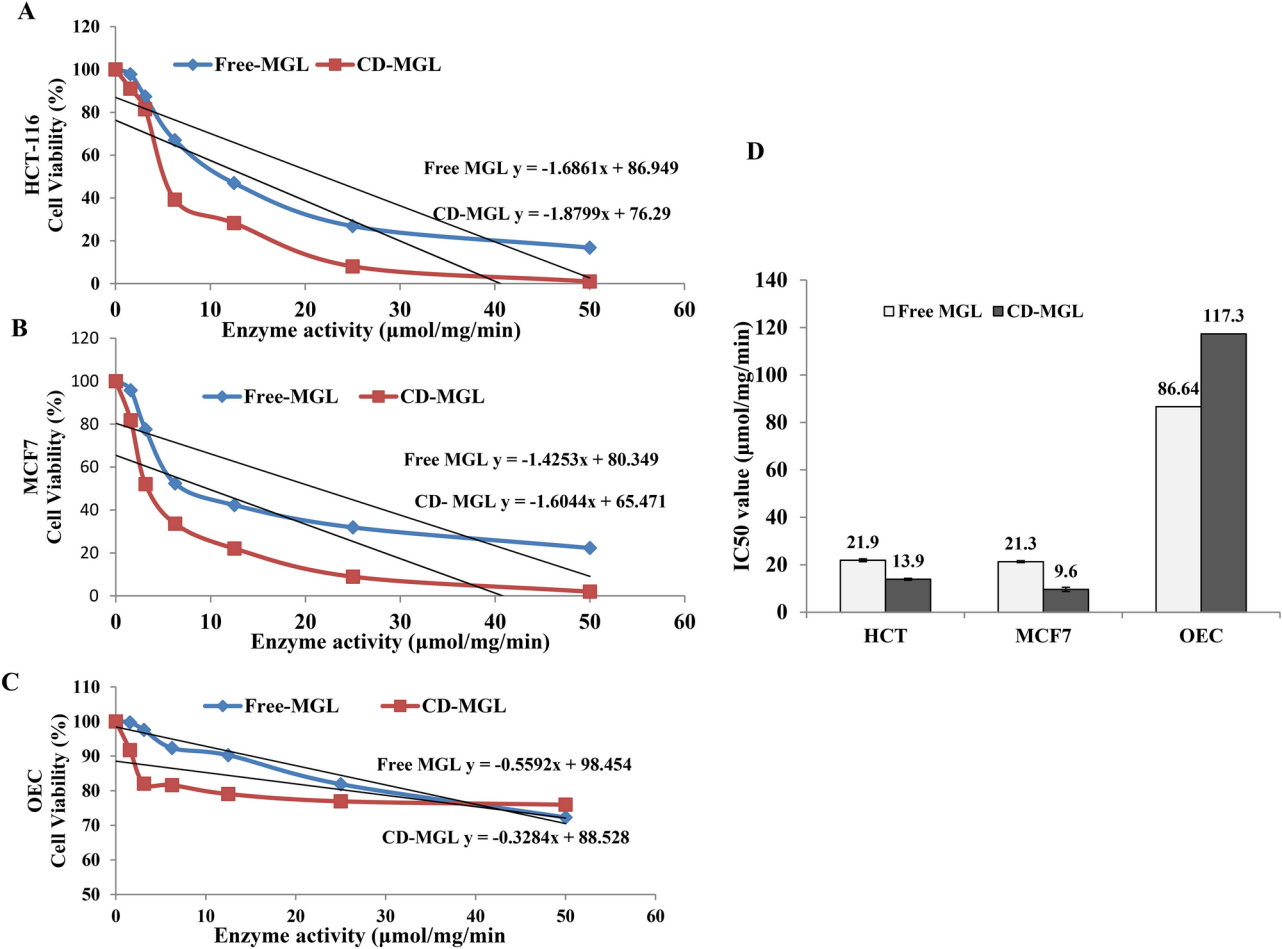
Antiproliferative activity of the free and CD-MGL against the different cell lines in vitro. Viability of the HCT-116 (**A**), and MCF7 (**B**), and OEC (**C**) cell lines n response to the free and CD-MGL conjugates. (**D**), The IC50 values of the free and CDMGL towards the HCT16, MCF7 and OEC cell lines.

The antiproliferative activity of the native and CD-MGL conjugates towards the breast carcinoma (MCF7) and colon cancer cells lines (HCT116) was evaluated normalizing to the Oral Epithelial cells (OEC) as control. From the results (Fig. 6), the IC_50_ values of the free MGL for HCT116 and MCF7 cells were 21.9–21.3 μg/mg/min, with selectivity index about 4.1. For the CD-MGL, the IC_50_ values towards the HCT116 and MCF7 were 13.9 μg/mg/min and 9.6 μg/mg/min, with selectivity index 8.5 and 12.2, respectively, ensuring the enhanced activity of the enzyme upon cyclodextrin conjugation. The IC_50_ values of the free MGL and CD-MGL towards the OEC was 86.6 and 117.3 μg/mg/min, respectively. From the IC_50_ values of the enzymes, the activity of MGL against the tested cell lines was significantly increased upon cyclodextrin conjugation (Fig. 6C). So, the in vitro antiproliferative activity of the MGL to hydrolyze L-methionine, with subsequent ceasing the growth of the tumor cells was strongly enhanced upon cyclodextrin conjugation, as revealed from the cellular viability. Overall, the in vitro antiproliferative activity of MGL was increased by ~ 1.6 folds upon cyclodextrin conjugation.

### Cytotoxicity and anticancer activity of native and CD-MGL in vivo

**Fig. 7:**
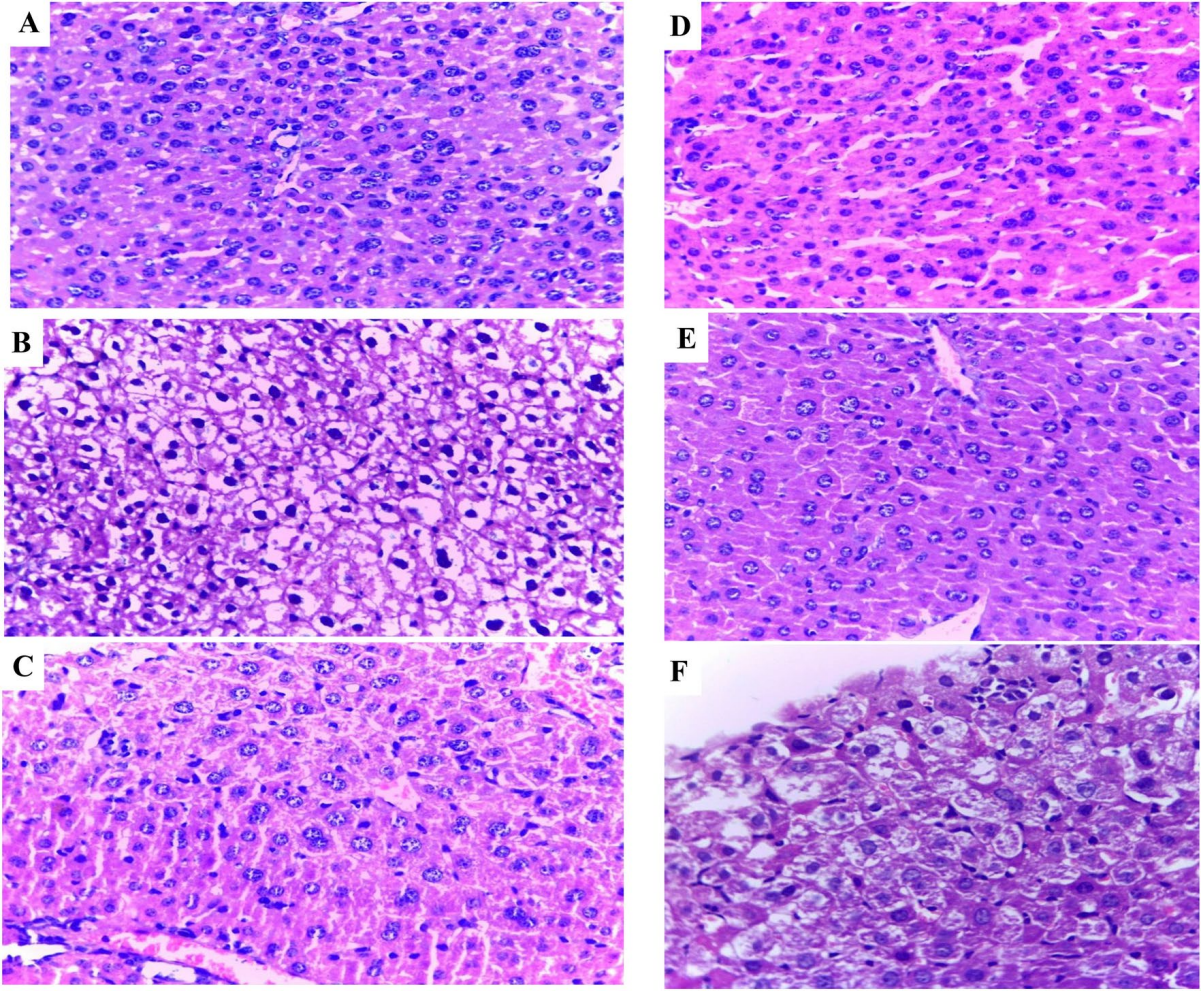
Histopathological sections of liver control and Ehrlich ascites carcinoma bearing mice in response to treatment with the free and CD-MGL conjugates at 400 × by Olympus BX51 Microscope. A, Liver sections of negative control mice. B, Liver sections of mice with Ehrlich Ascites Carcinoma without enzymes treatment. C, Liver sections of normal mice without tumors with the free MGL. D, Liver sections of mice with Ehrlich tumors treated with the free MGL. E, liver sections of normal mice treated with CD-MGL. F, liver sections of mice with Ehrlich tumors treated with CD-MGL.

The cytotoxicity and anticancer activity of the free and CD-MGL conjugates were assessed in Albino female in vivo. From the histopathological sections of the negative control (Fig. 7A), the section of liver appears with normal architecture, and central vein arranged normal hepatocyte around it in cords with bright eosinophilic cystoplasm. While, the liver sections of mice with Ehrlich Ascites carcinoma without enzymes treatment, exhibited a strong hepatocellular carcinoma with complete loss of normal liver architecture with diffuse infiltrate of malignant cells, with an obvious polymorphism and hyperchromatism, abnormal mitosis, most of cells showed necrosis with high proliferation and bizarre giant cells (Fig. 7B). The histopathological analysis of liver of normal mice “without tumors” with the free MGL, reveals the central vein with surrounding normal hepatocytes arranged in cords with bright eosinophilic, the periphery of the section reveal localized area of cloudy swelling cytoplasm with shrinking nucleus (Fig. 7C). However, in response to treatment with free MGL and CD-MGL, the liver sections of mice with Ehrlich tumors showed congested blood vessel and hepatocyte regeneration arranged in cords with multi-nucleation (Fig. 7D, E,F).

The toxicity of free and CD-MGL conjugates in mice was assessed based on the biochemical parameters of mice injected with single enzyme dose. From the biochemical profile (Table S4), the activity of liver enzymes ALT, AST, in addition to urea and creatinine concentrations were strongly negatively affected in negative and positive controls. However, in response to the treatment with the free and CD-MGL conjugates, the activity of ALT and AST were greatly improved. In positive control of Ehrlich tumors bearing mice (without enzymes treatment), the levels of the tested liver enzymes (AST, ALT) were dramatically increased by ~ 4.0 folds compared to the negative control animals. However, upon treatment by the free and CD-MGL conjugates, the activity of the AST and ALT were greatly alleviated ensures the powerful antiproliferative activity of the enzymes preparations in vivo*.* As well as, the concentration of creatinine in mice with Ehrlich tumors were strongly increased by about 3 folds (1.5 mg/dl), however, upon treatment with the enzymes, the titer of creatinine was restored to the same level of control (~ 0.4 mg/dl). Interestingly, the antiproliferative activity of the MGL was greatly improved with cyclodextrin conjugation, as revealed from the in vivo biochemical properties, with no signs of toxicity on the experimental animals.

**Fig. 8:**
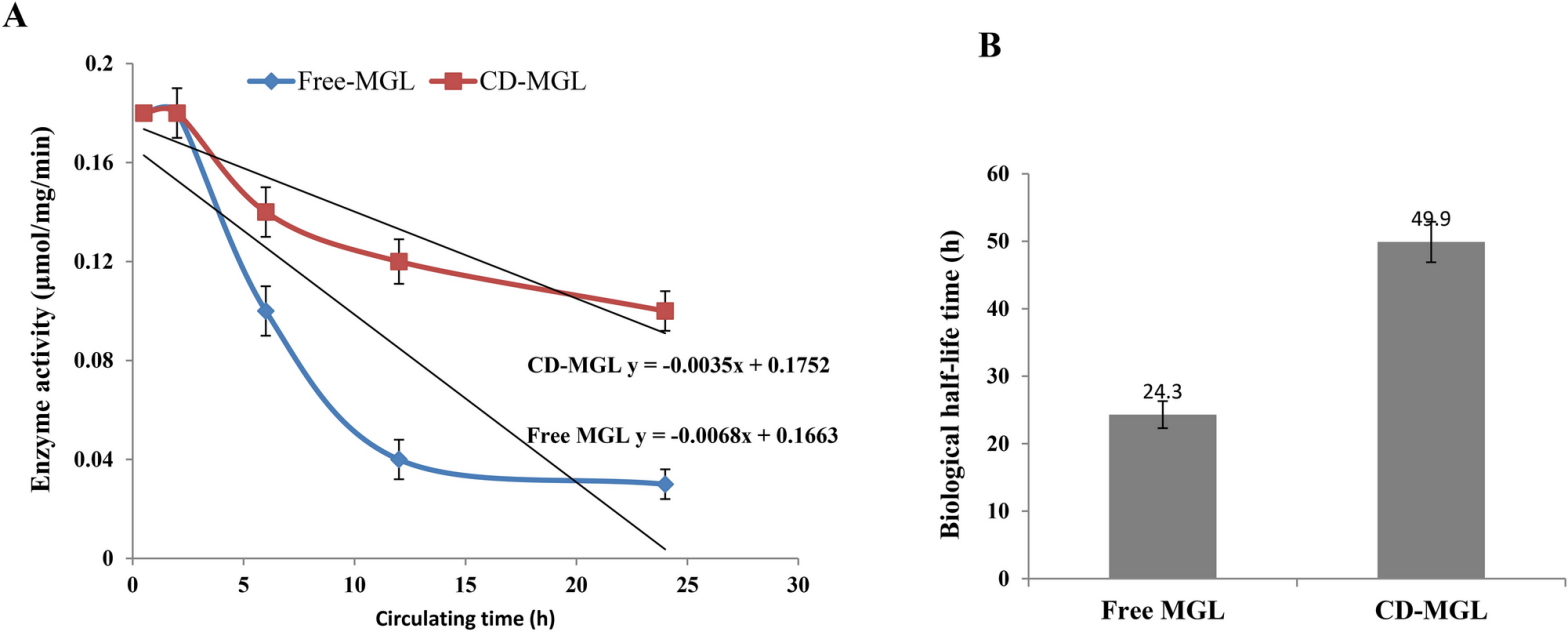
The pharmacokinetics properties of the purified A. flavipes free MGL and CD-MGL, in vivo, in BABL/c albino female mice were determined. Single dose (38 μmol/mg/min) for enzymes were intravenously injected to the mice in five replicate groups. The blood samples were collected intervally after 0.5, 2, 6, 12 and 24 h, and the enzymes activities (**A**) and the biological half-life time (**B**) of the free and CD-MGL were measured

The pharmacokinetic properties of the free and CD-MGL were determined in vivo in mice after single dosing. The blood samples were collected intervally, the enzyme activity was determined after different circulating time. From the results (Fig. 8), the activity of the free MGL was significantly reduced, compared to the CD-MGL, ensuring the protective role of CD on enzyme conformational and catalytic structures. The biological half-life of the free and CD-MGL was 24.3 and 49.9 h, respectively, i.e. by about two folds structural stability increment of MGL upon using cyclodextrin.

## Discussion

L-Methionine γ-lyase has been authenticated as one of the most promising targeted-therapy against various types of solid tumors^15,51,52^. The methionine addiction (Hoffman Effect) of tumor cells has been metabolically authenticated extensively, that mainly due to the absence of methionine synthase of tumor cells, making their absolute dependence on plasma L-methionine for rapid growth, unlike to the normal cell that designated as plasma methionine-independent^5,12,51^. The MGL has been purified and characterized from *Aspergillus flavipes,* displayed a powerful activity against various tumor cell lines in vitro, as well as, promising pharmacokinetic properties in vivo^17–21^. The structural stability, catalytic properties and anticancer activity of *A. flavipes* MGL were strongly improved upon chemical modification with polyethylene glycol (PEGylation), and dextran conjugation^17–21^. However, *A. flavipes* MGL denaturation, low immobilization yield, and catalytic efficiency of the enzyme that might be due to the chemical interaction during the immobilization process with these polymers are the major challenges that halt the further clinical implementation of this enzyme. Thus, the objective of this study was to increase the structural stability and catalytic efficiency of the purified *A. flavipes* MGL via conjugation with β-cyclodextrin “host–guest chemistry” without using any chemical catalysis, or cross-linkers, in addition to evaluate their anticancer activity in vitro and in vivo*.*

The lower productivity of MGL by *A. flavipes* is one of the technical challenges for large-scale production as revealed from our previous studies^17–21^. So, the productivity of MGL by *A. flavipes* has been optimized by Plackett–Burman design by evaluating the influence of the different independent variables of carbon, nitrogen and different growth factors. Upon Plackett–Burman nutritional screening, the yield of MGL by *A. flavipes* were increased by about two folds (12 μmol/mg/min), compared to the control medium. Similar results were conducted for maximizing the productivity of cytosine deaminase, tyrosinase, L-asparaginase^32,35,36,53,54^, in addition to various secondary metabolites by fungi^23,32,53,55–57^. The fungus was grown on the optimized media, and the intracellular crude proteins were extracted, and purified. By gel-filtration and ion-exchange chromatography, the enzyme was purified by about 3.2 folds with yield 82.3% comparing to the crude enzyme. The purified MGL had a molecular subunit structure of about 48 kDa under denaturing-PAGE. The enzyme was purified according to our previous design purification protocols to its molecular homogeneity, with the same molecular mass and subunit structure^32,33,38,39,46,58,59^.

The purified *A. flavipes* MGL was conjugated with β-cyclodextrin at different molar ratios, the highest immobilization yield of MGL with β-cyclodextrin (80%) was obtained at 100:1 of MGL/ β-cyclodextrin. The higher immobilization yield of MGL with β-cyclodextrin is one of the most affordable criterion over the other conjugation methods that might be due to the complete lack of chemical catalysts or cross-linkers, which might had a negatively effect on the enzyme catalytic properties. The activity of CD-MGL being higher than the activity of *A. flavipes* MGL conjugated with dextran and polyethylene glycol^17,21,26,32^. The higher affordable activity CD-MGL elaborates from the unique mechanism of conjugation “host–guest conjugation”^28,29^. Recently, β-cyclodextrin has been recognized as one of the most sophisticated cyclic oligosaccharides for delivery of therapeutic enzymes and bioactive metabolites^28,29^. The degree of conjugation of MGL with β-cyclodextrin was assessed based on the frequency of the total surface reactive amino groups of MGL, the reactivity of these surface amino groups were reduced by ~ 45% with β-cyclodextrin conjugation, ensuring the interactions of the enzyme surface amino groups with β-cyclodextrin. From the modeling analysis, the MGL binds with the cyclodextrin by various non-covalent, hydrogen bonding, Van der Waals forces interactions of the surface aromatic amino acids “tyrosine, phenylalanine, tryptophan”, in addition to serine, asparagine, alanine, and glutamic acid and hydroxyl groups of the cyclodextrins. Consistently, reduction of the initial activity of enzyme during the chemical conjugation process with dextran was reported for glucose oxidase^60^ and proteases^61^. Similarly, reduction to the activity of *A. flavipes* MGL and cystathionine γ-lyase upon covalent modification with polyethylene glycol was noticed^17,34,50^. The chemical interactions of the MGL and β-cyclodextrin were evaluated by FTIR. The MGL has a broad FTIR characteristic band centered at ~ 3400 cm^−1^ followed by low intensity band at ~ 2900 cm^−1^, that mainly arises from the stretching of hydroxyl groups and CH_2_ groups. A slight shift on the surface hydroxyl groups at 3435 cm^−1^ of MGL upon β-cyclodextrin conjugation was observed, revels the interaction of the surface hydroxyl groups with the glucose units of β-cyclodextrin. For the CD-MGL, a band centered at 1630 cm^−1^ was developed reveals the bending vibration of O–H bonds of adsorbed water molecules on the cyclodextrin surface. The interaction of the surface aromatic amino acids “tryptophan, phenylalanine and tyrosine” with in the cavity of β-cyclodextrin has been reported frequently^28,29^. The CD-MGL conjugate displayed an obvious resistance to proteolysis with proteinase K “serine protease of broad activity” and trypsin, ensuring the shielding of the proteolytic recognition sites on MGL surface, approving the therapeutic affordability in vivo. Similar results of MGL protection against the proteolytic cleavage with trypsin and various proteases were reported^62,63^. So, the strong hydrolysis of MGL with proteinase K being reasonable since this protease has non-specific, broad range activity, attacking multiple recognition sites, on contrary to the obvious resistance to proteolysis of MGL upon β-cyclodextrin conjugation.

The free and CD-MGL conjugates have the same activity pattern to the reaction temperature at 37–40 °C, reaction pH at 7.5, and pH stability at 6.5–8.0, ensuring the lack of interaction of cyclodextrin with the charged catalytic surface catalytic residues of MGL^17,23,33,39,50,64^. The putative pH precipitation (*p*I) of the free and CD-MGL were 5.0 and 6.0, respectively, confirming the higher solubility, resistance to denaturation and structural robustness in response to pHs, acquired by cyclodextrin conjugation. The higher solubility and shift of the *pI* of MGL upon cyclodextrin conjugation might be attributed to the interaction of the surface hydrophobic aromatic amino acids with the core of cyclodextrin by host–guest chemistry, unlike to the unbounded free aromatic amino acids “hydrophobic” on the surface of free MGL. The stability of the CD-MGL conjugates at 4 °C, as revealed from the half-life times (*T*_*1/2*_) were increased by about two-folds (16.2 days), compared to the free one, ensuring the acquired conformational stability of the enzyme upon cyclodextrin conjugation. The putative melting temperature (*T*_*m*_) of MGL was increased by about 2 folds (140 °C), upon cyclodextrin conjugation.

A dramatic stability of CD-MGL in response to the amino acids analogues “hydroxylamine, PMSF, DTNB, MBTH, and guanidine thiocyanate”, reveals the stabilizing effect of cyclodextrin to the enzyme by making a shell around the enzyme, via binding of the aromatic amino acids, thus shielding the surface cysteine, glutamine, asparagine and lysine residues to be accessible for the chemical inhibitors. Similar results were observed for the MGL conjugated with PEG residues and dextran polymer^17,21,23,32,50^. The free and CD-MGL conjugates have the same catalytic affinity towards the sulfur-containing amino acids as substrates ensures the lack of chemical interactions of cyclodextrin with the enzyme catalytic domains and tertiary structure of MGL. The catalytic affinity (*K*_M_) and catalytic efficiency (*K*_cat_*/K*_M_) of MGL was increased by two folds upon cyclodextrin conjugation, ensuring the re-orientation and re-shaping of the enzyme surface active sites. The higher affinity of MGL towards L-methionine as substrate upon cyclodextrin conjugation could be attributed to multiple interactions of L-methionine by various hydrogen bonds, and hydrophobic interactions.

The in vitro antiproliferative of MGL towards the HCT116 and MCF7 cells was increased by ~ 1.6 folds upon cyclodextrin conjugation as revealed from their IC_50_ values. The cytotoxicity and anticancer activity of the enzymes were assessed in vivo. In response to the treatment with the free and CD-MGL, the liver sections with Ehrlich tumors showed congested blood vessel and hepatocyte regeneration arranged in cords with multi-nucleation. Upon treatment with the free and CD-MGL, the activity of AST and ALT were improved, ensuring the powerful e activity of these enzymes preparations in vivo*.* Interestingly, the antiproliferative activity of the MGL was greatly improved with cyclodextrin conjugation, as revealed from the in vitro and in vivo biochemical properties, with no signs of toxicity on the experimental animals.

In conclusion, the productivity of MGL from *A. flavipes* was optimized, the enzyme was purified and chemically conjugated with β-cyclodextrin. The possible mechanistics of interactions of MGL with β-cyclodextrin was validated from the surface reactive residues by the molecular docking analysis. The biochemical and kinetic properties of the free enzyme and CD-MGL conjugates were comparatively characterized. The catalytic stability and kinetic properties of MGL towards L-methionine was strongly increased by about 2 folds, upon cyclodextrin conjugation. The in vitro activity of MGL against the HCT-116 and MC7 was increased by about 2 folds upon cyclodextrin conjugation. This is the first report, describing the conjugation of MGL with β-cyclodextrin, displaying an affordable structural and kinetic stability, in addition to the improved anticancer activity in vitro and in vivo*.* Further studies are ongoing to explore the pharmacokinetic properties of the free and CD-MGL, depletion of plasma L-methionine, and Coenzyme (PLP) stability, in vivo.

## Supplementary Information

Supplementary Information 1.

Supplementary Information 2.

## Data Availability

Data is provided within the manuscript or supplementary information files.
