# Supplementary material for: Aspergillus flavipes L-methionine γ-lyase-β-cyclodextrin conjugates with improved stability, catalytic efficiency and anticancer activity
Source: Sci Rep. 2024 Nov 12;14:27715. doi: 10.1038/s41598-024-78368-5 (PMC11557573; doi:10.1038/s41598-024-78368-5)
Supplement: Supplementary file 1 — Supplementary Information 1. [file 41598_2024_78368_MOESM1_ESM.docx]

**Table S1. Independent variables at two levels for MGL production by *Aspergillus flavipes* by Plackett-Burman design**

| **Code** | **Variables** | **level -1** | **level 1** |
| --- | --- | --- | --- |
| X1 | Methionine (g/L) | 2 | 4 |
| X2 | Glycine (g/L) | 2 | 4 |
| X3 | Valine (g/L) | 2 | 4 |
| X4 | L-Asparagine (g/L) | 2 | 4 |
| X5 | L-Glutamine (g/L) | 2 | 4 |
| X6 | L-Lysine (g/L) | 2 | 4 |
| X7 | D-Glucose (g/L) | 2 | 10 |
| X8 | Fructose (g/L) | 2 | 8 |
| X9 | Xylose (g/L) | 2 | 8 |
| X10 | Lactose (g/L) | 1 | 4 |
| X11 | Sucrose (g/L) | 2 | 8 |
| X12 | MgCL2 (g/L) | 0.1 | 0.4 |
| X13 | ZnCl2 (g/L) | 0.1 | 0.4 |
| X14 | CaCl2 (g/L) | 0.1 | 0.4 |
| X15 | NaCl | 0.1 | 0.4 |
| X16 | Vitamin B6 (g/L) | 0.2 | 0.6 |
| X17 | pH | 6.0 | 8.0 |
| X18 | Incubation time | 7 | 12 |
| X19 | Shacking Speed | 50 | 150 |

**Table S2.** ANOVA for selected factorial model Analysis of variance table [Partial sum of squares - Type III]. ANOVA for selected factorial model,

|  | Sum of |  | Mean | F | p-value |  |
| --- | --- | --- | --- | --- | --- | --- |
| Source | Squares | df | Square | Value | Prob > F |  |
| Model | 686.1 | 8 | 85.76 | 19.12 | < 0.0001 | significant |
| B-Glycine | 59.08 | 1 | 59.08 | 13.17 | 0.004 |  |
| G-Glucose | 316.34 | 1 | 316.34 | 70.52 | < 0.0001 |  |
| H-Fructose | 31.19 | 1 | 31.19 | 6.95 | 0.0231 |  |
| K-Lactose | 70.53 | 1 | 70.53 | 15.72 | 0.0022 |  |
| M-MgCl_2_ | 35.22 | 1 | 35.22 | 7.85 | 0.0172 |  |
| O-CaCl_2_ | 91.98 | 1 | 91.98 | 20.51 | 0.0009 |  |
| P-Vitamin B6 | 44.78 | 1 | 44.78 | 9.98 | 0.0091 |  |
| Q-Vitamin B12 | 36.97 | 1 | 36.97 | 8.24 | 0.0152 |  |
| Residual | 49.34 | 11 | 4.49 |  |  |  |
| Cor Total | 735.44 | 19 |  |  |  |  |

Table S3. Substrate affinity of the purified MGL and CD-MGL conjugates.

|  | Free MGL | | CD-MGL | |
| --- | --- | --- | --- | --- |
|  | DTNB Assay | Nessler's assay | DTNB Assay | Nessler's assay |
| L-Methionine | 100 | 100 | 100 | 100 |
| DL-Homocysteine | 94 | 96 | 95 | 96 |
| L-Cystine | 4 | 10 | 6 | 7 |
| L-Cysteine | 15 | 20 | 10 | 18 |
| L-Lysine | 0 | 10 | 0 | 12 |
| DL-Asparagine | 0 | 8 | 0 | 10 |
| L-Tyrosine | 0 | 12 | 0 | 14 |
| D-Glycine | 0 | 14 | 0 | 16 |
| DL-Phenylalanine | 0 | 7 | 0 | 15 |
| L-Alanine | 0 | 6 | 0 | 14 |
| L-Glutamine | 0 | 12 | 0 | 12 |

**Table S4. Biochemical parameters of mice in response to free and CD-MGL conjugates.**

| Sample | **AST**  **(U)** | **ALT (U)** | **Urea (mg/dl)** | **Creatinine (mg/dl)** |
| --- | --- | --- | --- | --- |
| Negative Control | **33.5±3.2** | **42± 4.1** | **47± 3.2** | **0.595±0.09** |
| Positive control (Without treatment) | **139± 6.8** | **171.5± 11..3** | **99.5±8.7** | **1.5±0.2** |
| ER treated with free MGL | **72±4.9** | **83±7.2** | **70±6.3** | **0.41±0.08** |
| ER treated with CD-MGL | **68±5.1** | **80.5± 6.9** | **63.5±5.1** | **0.425±0.09** |
