# Supplementary material for: Aspergillus flavipes L-methionine γ-lyase-β-cyclodextrin conjugates with improved stability, catalytic efficiency and anticancer activity
Source: Sci Rep. 2024 Nov 12;14:27715. doi: 10.1038/s41598-024-78368-5 (PMC11557573; doi:10.1038/s41598-024-78368-5)
Supplement: Supplementary file 2 — Supplementary Information 2. [file 41598_2024_78368_MOESM2_ESM.pptx]

## Slide 1
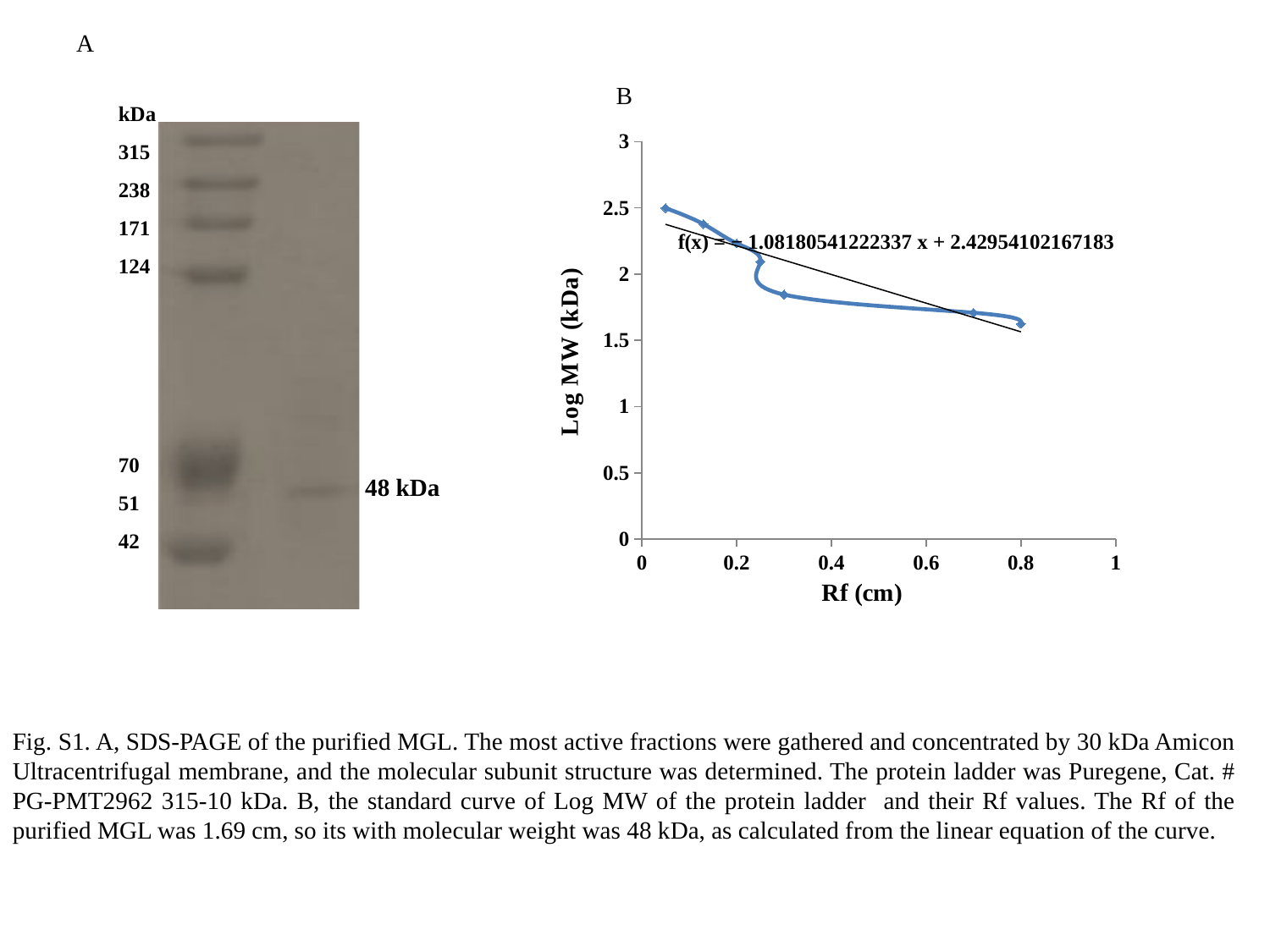

A
B
### Chart
| Category | Log MW |
|---|---|kDa
315
238
171
124
70
51
42
 48 kDa
Fig. S1. A, SDS-PAGE of the purified MGL. The most active fractions were gathered and concentrated by 30 kDa Amicon Ultracentrifugal membrane, and the molecular subunit structure was determined. The protein ladder was Puregene, Cat. # PG‐PMT2962 315‐10 kDa. B, the standard curve of Log MW of the protein ladder and their Rf values. The Rf of the purified MGL was 1.69 cm, so its with molecular weight was 48 kDa, as calculated from the linear equation of the curve.

## Slide 2
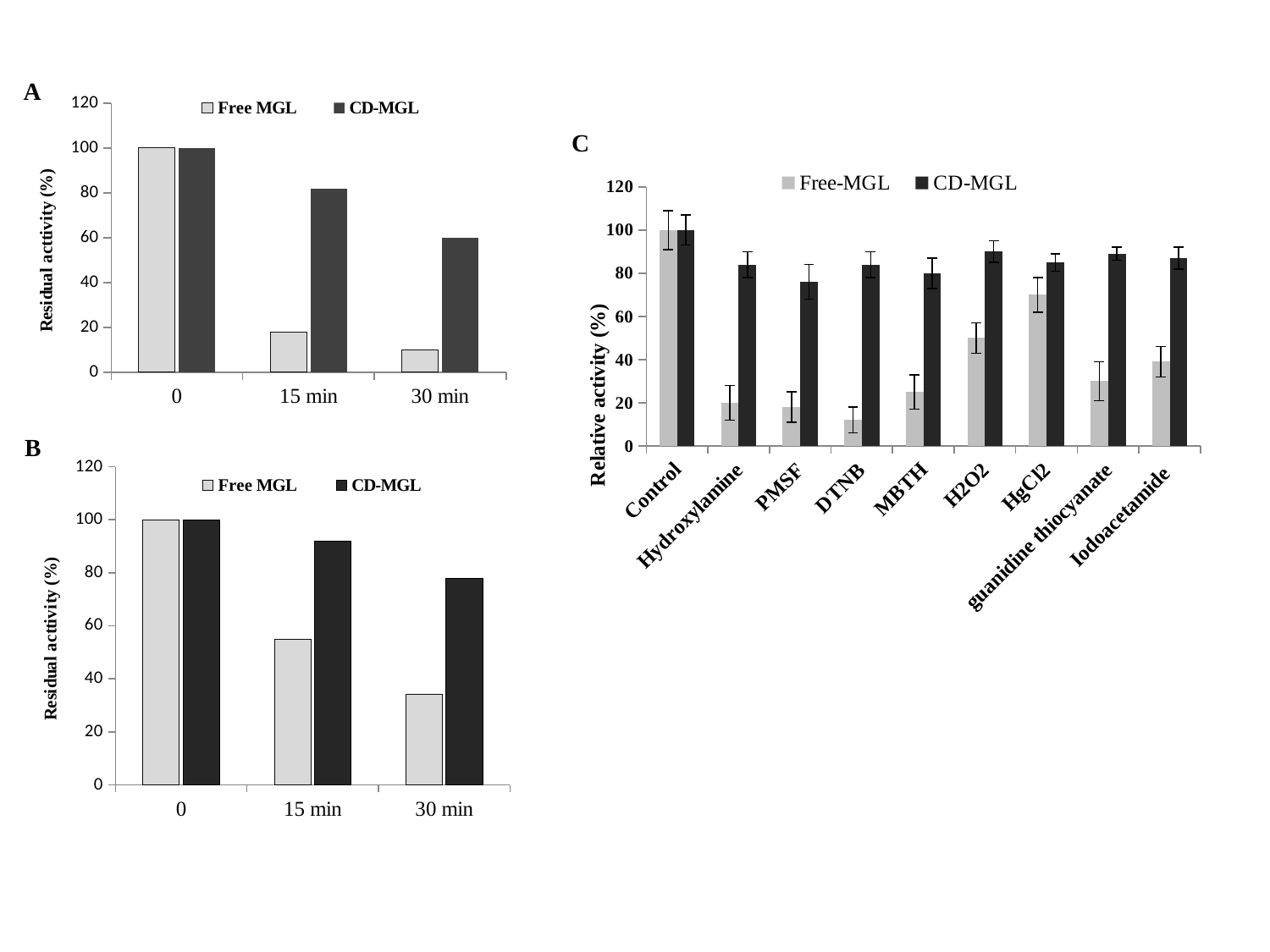

A
### Chart
| Category | Free MGL | CD-MGL |
|---|---|---|
| 0 | 100.0 | 100.0 |
| 15 min | 18.0 | 82.0 |
| 30 min | 10.0 | 60.0 |C
### Chart
| Category | Free-MGL | CD-MGL |
|---|---|---|
| Control | 100.0 | 100.0 |
| Hydroxylamine | 20.0 | 84.0 |
| PMSF | 18.0 | 76.0 |
| DTNB | 12.0 | 84.0 |
| MBTH | 25.0 | 80.0 |
| H2O2 | 50.0 | 90.0 |
| HgCl2 | 70.0 | 85.0 |
| guanidine thiocyanate | 30.0 | 89.0 |
| Iodoacetamide | 39.0 | 87.0 |B
### Chart
| Category | Free MGL | CD-MGL |
|---|---|---|
| 0 | 100.0 | 100.0 |
| 15 min | 55.0 | 92.0 |
| 30 min | 34.0 | 78.0 |

## Slide 3
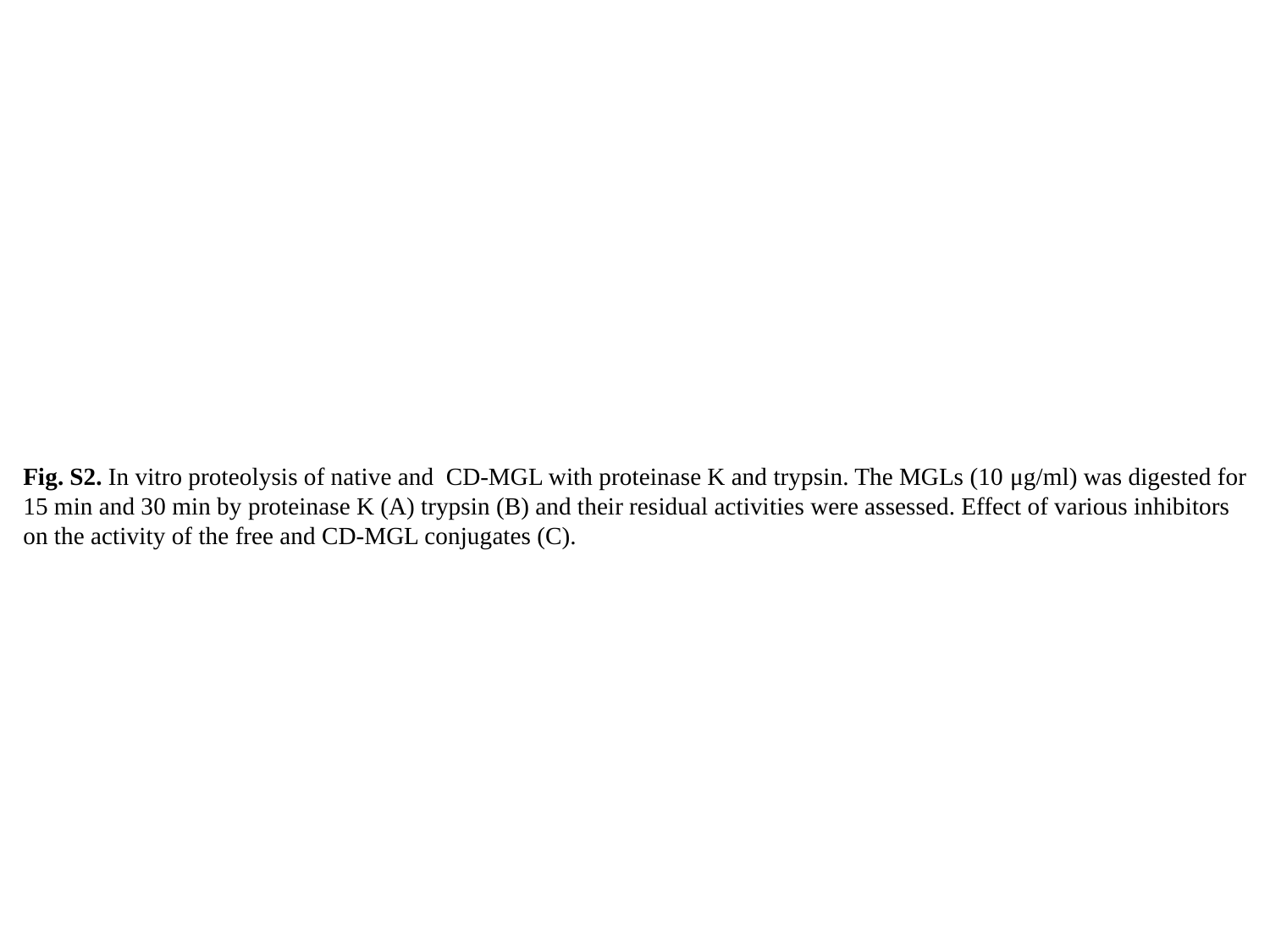

Fig. S2. In vitro proteolysis of native and CD-MGL with proteinase K and trypsin. The MGLs (10 μg/ml) was digested for 15 min and 30 min by proteinase K (A) trypsin (B) and their residual activities were assessed. Effect of various inhibitors on the activity of the free and CD-MGL conjugates (C).

## Slide 4
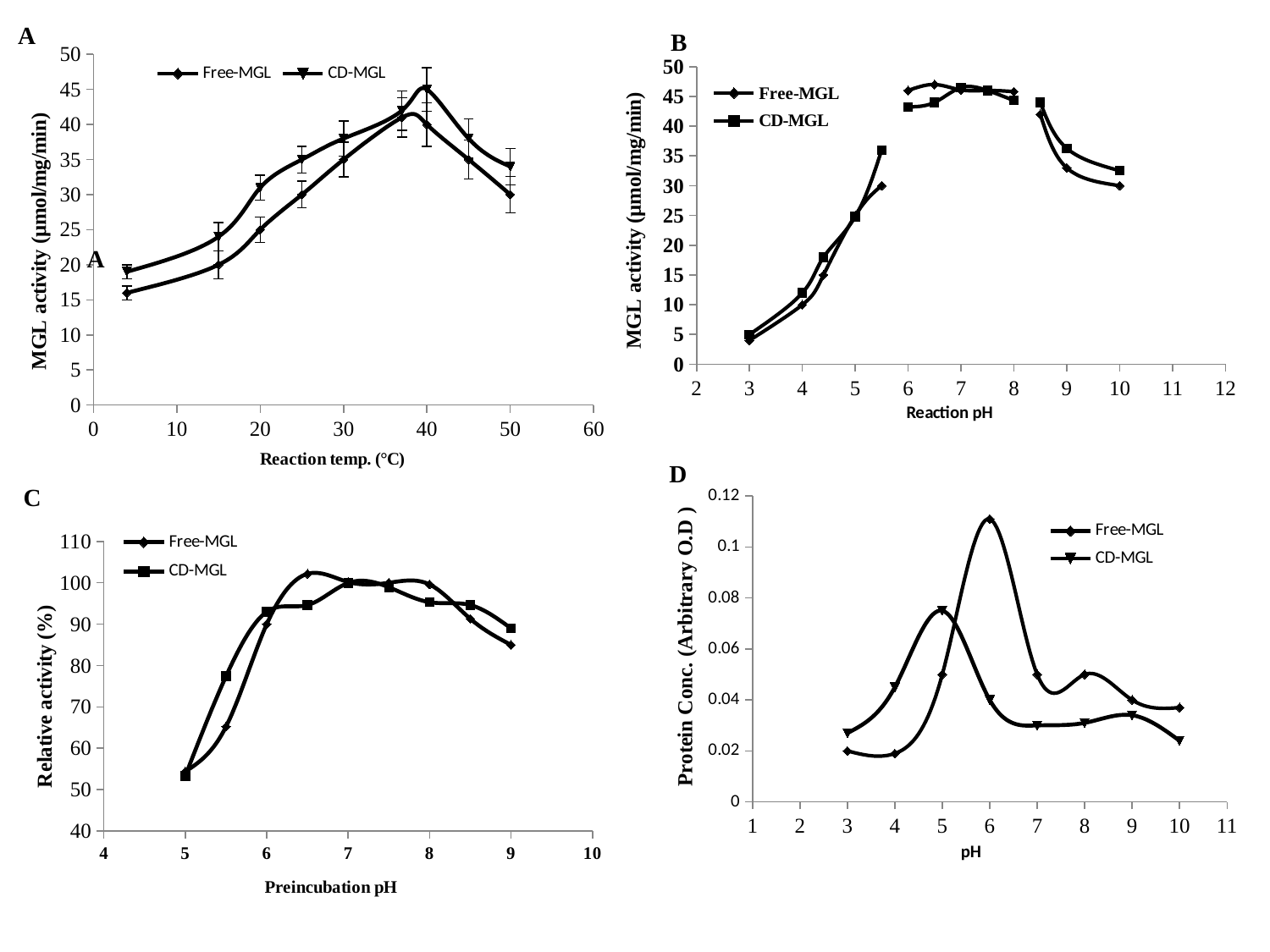

A
B
### Chart
| Category | Free-MGL | CD-MGL |
|---|---|---|
### Chart
| Category | Free-MGL | CD-MGL |
|---|---|---|A
D
C
### Chart
| Category | Free-MGL | CD-MGL |
|---|---|---|
### Chart
| Category | Free-MGL | CD-MGL |
|---|---|---|

## Slide 5
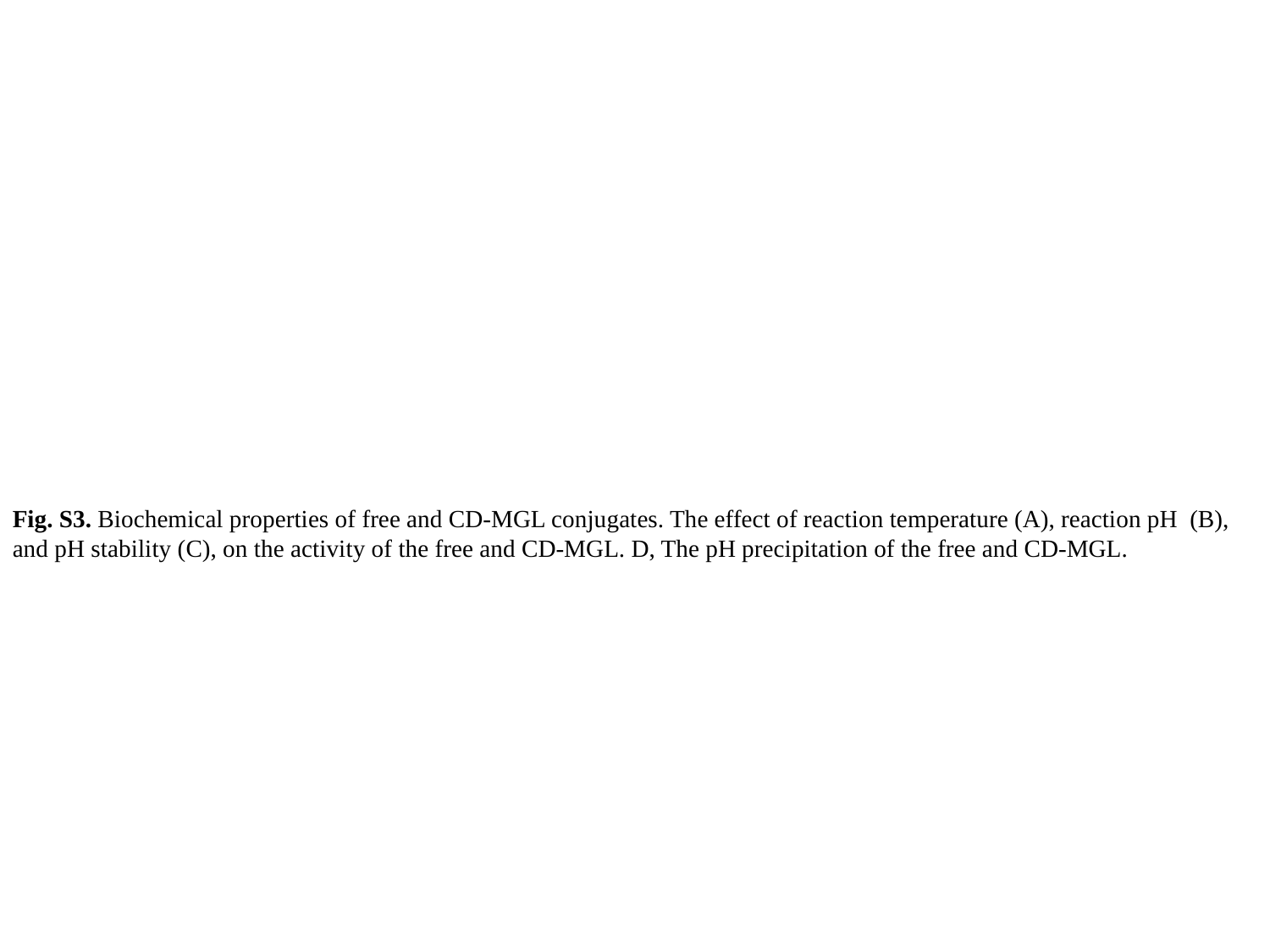

Fig. S3. Biochemical properties of free and CD-MGL conjugates. The effect of reaction temperature (A), reaction pH (B), and pH stability (C), on the activity of the free and CD-MGL. D, The pH precipitation of the free and CD-MGL.

## Slide 6
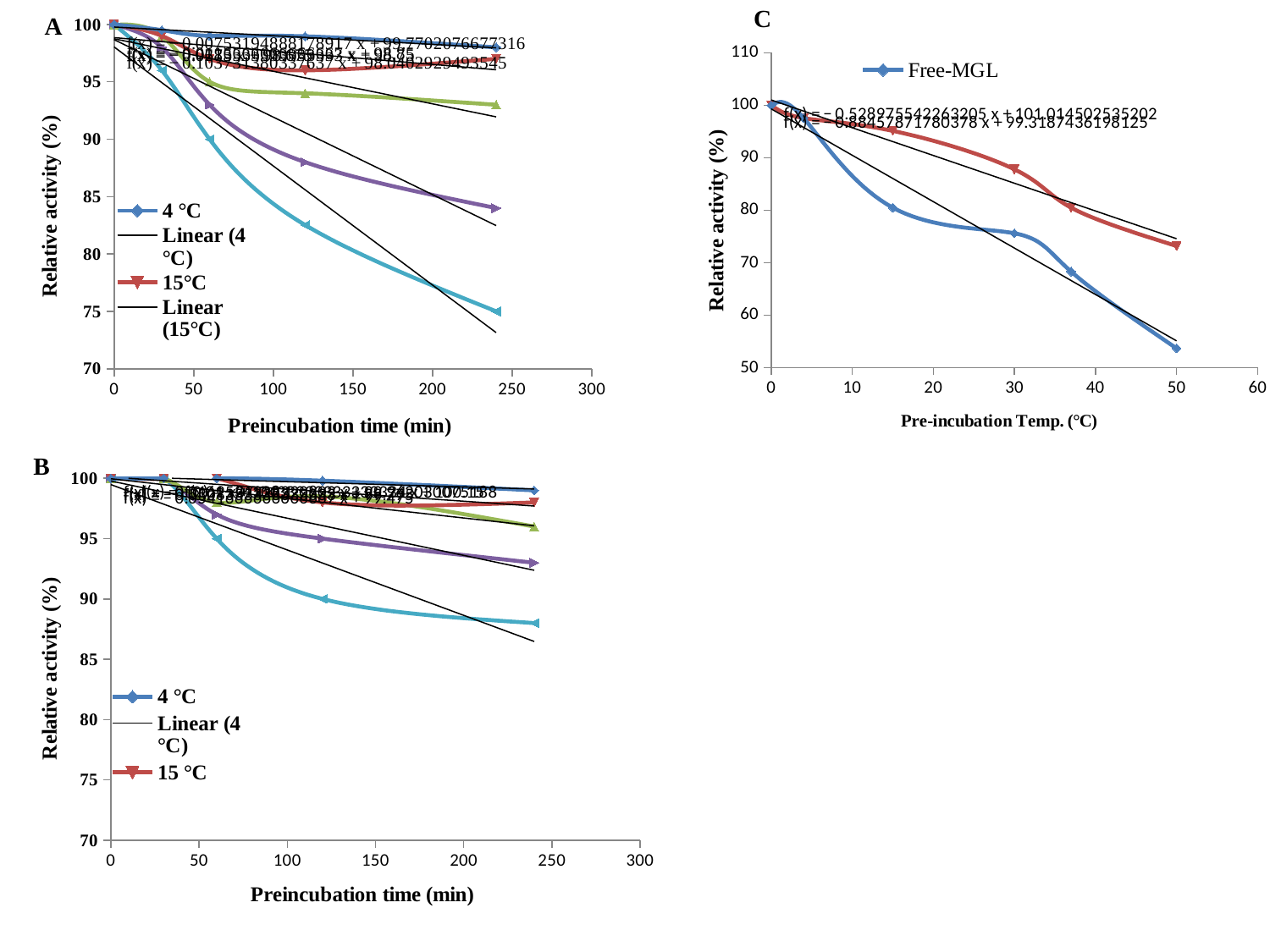

C
### Chart
| Category | 4 °C | 15°C | 30°C | 37 °C | 50 °C |
|---|---|---|---|---|---|A
### Chart
| Category | Free-MGL | CD-MGL |
|---|---|---|B
### Chart
| Category | 4 °C | 15 °C | 30°C | 37°C | 50°C |
|---|---|---|---|---|---|

## Slide 7
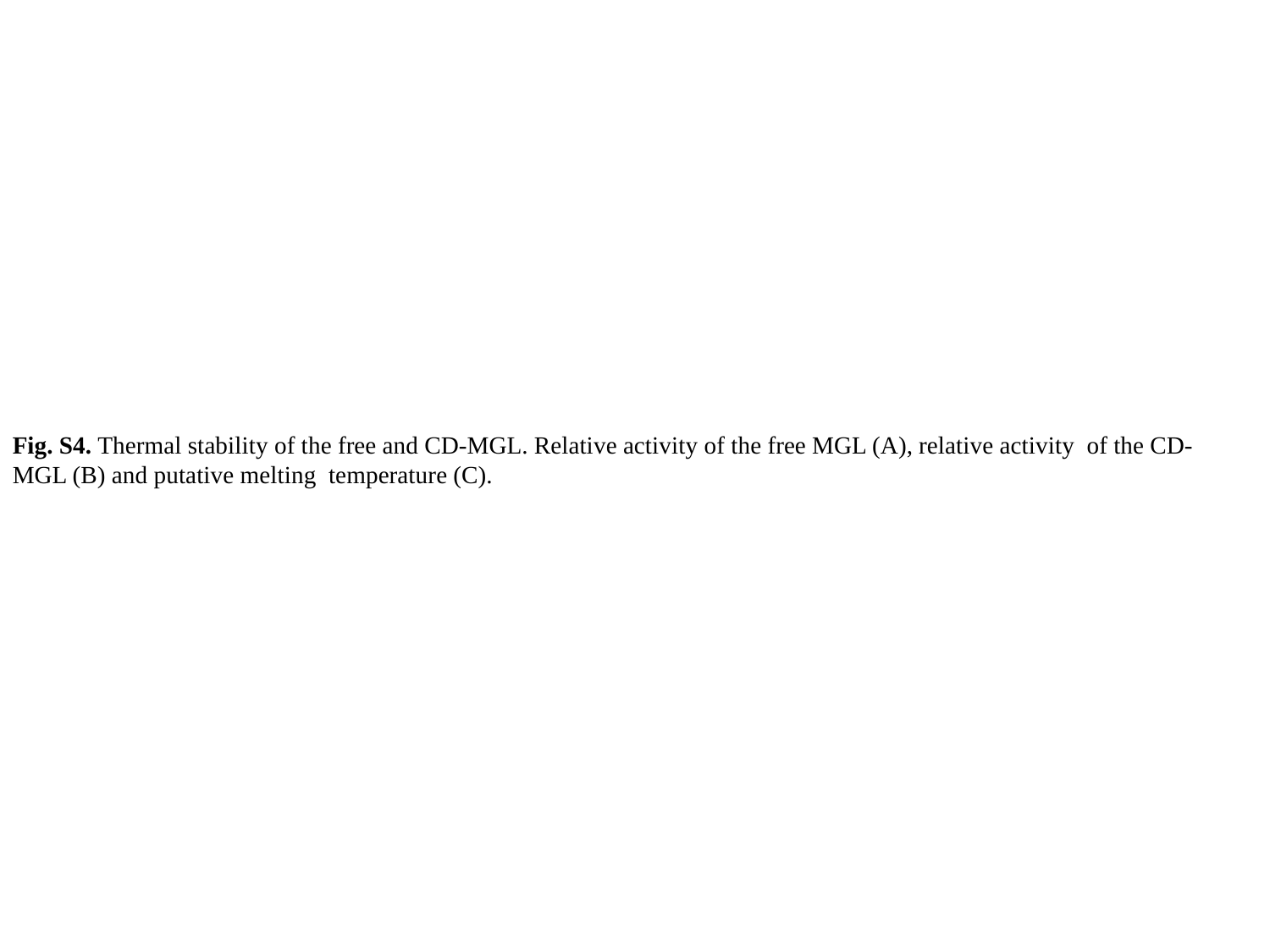

Fig. S4. Thermal stability of the free and CD-MGL. Relative activity of the free MGL (A), relative activity of the CD-MGL (B) and putative melting temperature (C).
